# Does the use of higher versus lower oxygen concentration improve neurodevelopmental outcomes at 18–24 months in very low birthweight infants?

**DOI:** 10.1186/s13063-024-08080-2

**Published:** 2024-04-04

**Authors:** Georg M. Schmölzer, Elizabeth V. Asztalos, Marc Beltempo, Hector Boix, Eugene Dempsey, Walid El-Naggar, Neil N. Finer, Jo-Anna Hudson, Amit Mukerji, Brenda H. Y. Law, Maryna Yaskina, Prakesh S. Shah, Ayman Sheta, Amuchou Soraisham, William Tarnow-Mordi, Max Vento, behalf of the HiLo trial collaborators

**Affiliations:** 1 Centre for the Studies of Asphyxia and Resuscitation, Royal Alexandra Hospital, 10240 Kingsway Avenue NW, Edmonton, AB T5H 3V9 Canada; 2 Dept. of Pediatrics, University of Alberta, Edmonton, Canada; 3 Department of Newborn & Developmental Paediatrics, Sunnybrook Health Sciences Centre and University of Toronto, Toronto, ON Canada; 4 Departement of Pediatrics, Montreal Children’s HospitalMcGill University Health CenterMcGill University, Montreal, QC Canada; 5 Division of Neonatology, Dexeus Quironsalud University Hospital, Barcelona, Spain; 6 INFANT Research Centre, University College Cork, Cork, Ireland; 7 Department of Paediatrics, Dalhousie University, Halifax, Canada; 8 School of Medicine, University of California, San Diego, CA USA; 9 Sharp Mary Birch Hospital for Women and Newborns, San Diego, USA; 10 Faculty of Medicine, Memorial University of Newfoundland, St. John’s, NF Canada; 11 Division of Neonatology, Department of Pediatrics, McMaster University, Hamilton, ON Canada; 12 Women and Children’s Health Research Institute (WCHRI), University of Alberta, Edmonton, Canada; 13 Department of Pediatrics, Mount Sinai Hospital and University of Toronto, Toronto, Canada; 14 Department of Pediatrics and Child Health, University of Manitoba, Winnipeg, MB Canada; 15 Department of Pediatrics, Foothills Medical Centre, University of Calgary, Calgary, AB Canada; 16 Alberta Childrens Hospital Research Institute, University of Calgary, Alberta, Canada; 17 Trials Centre, National Health and Medical Research Council Clinical, University of Sydney, Camperdown, Australia; 18 Department of Pediatrics, La Fe University and Polytechnic Hospital, Valencia, Spain

**Keywords:** Infant, Extremely preterm, Neonatal intensive care, Delivery room, Oxygen, Neonatal mortality

## Abstract

**Background:**

Immediately after birth, the oxygen saturation is between 30 and 50%, which then increases to 85–95% within the first 10 min. Over the last 10 years, recommendations regarding the ideal level of the initial fraction of inspired oxygen (FiO_2_) for resuscitation in preterm infants have changed from 1.0, to room air to low levels of oxygen (< 0.3), up to moderate concentrations (0.3–0.65). This leaves clinicians in a challenging position, and a large multi-center international trial of sufficient sample size that is powered to look at safety outcomes such as mortality and adverse neurodevelopmental outcomes is required to provide the necessary evidence to guide clinical practice with confidence.

**Methods:**

An international cluster, cross-over randomized trial of initial FiO_2_ of 0.3 or 0.6 during neonatal resuscitation in preterm infants at birth to increase survival free of major neurodevelopmental outcomes at 18 and 24 months corrected age will be conducted. Preterm infants born between 23^0/7^ and 28^6/7^ weeks’ gestation will be eligible. Each participating hospital will be randomized to either an initial FiO_2_ concentration of either 0.3 or 0.6 to recruit for up to 12 months’ and then crossed over to the other concentration for up to 12 months. The intervention will be initial FiO_2_ of 0.6, and the comparator will be initial FiO_2_ of 0.3 during respiratory support in the delivery room. The sample size will be 1200 preterm infants. This will yield 80% power, assuming a type 1 error of 5% to detect a 25% reduction in relative risk of the primary outcome from 35 to 26.5%. The primary outcome will be a composite of all-cause mortality or the presence of a major neurodevelopmental outcome between 18 and 24 months corrected age. Secondary outcomes will include the components of the primary outcome (death, cerebral palsy, major developmental delay involving cognition, speech, visual, or hearing impairment) in addition to neonatal morbidities (severe brain injury, bronchopulmonary dysplasia; and severe retinopathy of prematurity).

**Discussion:**

The use of supplementary oxygen may be crucial but also potentially detrimental to preterm infants at birth. The HiLo trial is powered for the primary outcome and will address gaps in the evidence due to its pragmatic and inclusive design, targeting all extremely preterm infants. Should 60% initial oxygen concertation increase survival free of major neurodevelopmental outcomes at 18–24 months corrected age, without severe adverse effects, this readily available intervention could be introduced immediately into clinical practice.

**Trial registration:**

The trial was registered on January 31, 2019, at ClinicalTrials.gov with the Identifier: NCT03825835.

**Supplementary Information:**

The online version contains supplementary material available at 10.1186/s13063-024-08080-2.

## Introduction

### Background and rationale {6a}

#### Normal oxygen transition at birth

At birth, the oxygen saturation (SpO_2_) is around 30% [1], which then increases over the next 7–10 min to values of 85–95% [2]. The goal of a successful resuscitation for extremely low birth weight (ELBW) infants is to facilitate transition from intrauterine SpO_2_ levels to the accepted post-transitional neonatal range using simple SpO_2_ targets [3, 4].

#### Concerns for oxygen toxicity and deficit

The use of supplementary oxygen may be crucial but also potentially detrimental to preterm infants at birth. High oxygen levels may lead to organ damage through oxidative stress, while low oxygen levels may lead to increased mortality. Excess of oxygen free radicals in infants intrinsically deficient in enzymatic antioxidants and non-enzymatic antioxidants may contribute to these morbidities. Pulmonary oxygen toxicity, through the generation of reactive oxygen and nitrogen species in excess of antioxidant defenses, is believed to be a major contributor to the development of bronchopulmonary dysplasia (BPD) [5–10]. Varsila et al. noted that immaturity is the most important factor explaining free radical-mediated pulmonary protein oxidation in ELBW infants and that oxidation of proteins is related to the development of BPD [11]. Using lower oxygen concentrations at birth results in decreased oxidative stress markers and decreased risk of developing BPD compared to higher oxygen concentrations [12]. Other organs that may be damaged by such oxidative stress include kidneys, myocardium, and the retina [13–16].

#### The current International Liaison Committee on Resuscitation (ILCOR) guidelines

In 2010, ILCOR guidelines were revised to recommend that air or “less oxygen” should be used initially for preterm infants [17, 18] and that oxygen concentrations should be adjusted during resuscitation to meet SpO_2_ ranges that were similar to those of spontaneously breathing, healthy full-term infants [2]. However, the 2015 guidelines acknowledged the lack of evidence for either harm or benefit in starting resuscitation with either lower (< 0.3) or higher (> 0.65) FiO_2_ for preterm infants (i.e., < 37 weeks’ gestation) [19, 20] as well as the need for further research into establishing appropriate time-specific oxygen targets and to determine neurodevelopmental consequences of this practice in preterm infants. Indeed, a recent survey of 630 clinicians from 25 countries showed that the majority would initiate preterm infant delivery room stabilization with 0.30–0.4 initial FiO_2_ [21].

#### Summary of current evidence

Randomized controlled trials evaluating different oxygen concentrations for resuscitation of preterm infants have been occurring for the past 25 years [10, 22–31]. Furthermore, several systematic reviews highlight the ongoing concern regarding the knowledge gap in this area. Earlier systematic reviews of Brown et al. [32] and Saugstad et al. [33] found a significant (pooled risk ratio (RR) 0.65 [95% confidence interval (CI): 0.43, 0.98]) or borderline significant (relative risk for mortality 0.62 [95% CI: 0.37–1.04]) reduction in mortality when a low (21–50% or 21–30%) compared to a high (> 50% or ≥ 60%) oxygen concentration was used for initial stabilization in preterm infants (≤ 37 or ≤ 32 weeks), respectively. The most recent systematic review by Oei et al. [34] included trials with the same oxygen criteria as Saugstad et al. [33] as well as titration strategies to predefined SpO_2_ targets in preterm infants (≤ 28^+6^ weeks). No differences were found in the overall risk of death with either lower (≤ 30%) or higher (≥ 60%) oxygen concentrations. Collectively, while conclusions for each systematic review differed, all the authors emphasized the need for larger, well-designed trials to provide definitive recommendations for oxygen strategies during preterm delivery room resuscitation.

In a non-pre-specified analysis of the Targeted Oxygen in the Resuscitation of Preterm Infants Trial, infants of < 28 weeks’ gestation who received an initial FiO_2_ of 0.21 at resuscitation had higher hospital mortality than those given an initial FiO_2_ of 1.0 (10/46 [22%] vs. 3/54 [6%]; RR: 3.9 [95% CI: 1.1–13.4]; *p* = 0.01) [23]. Furthermore, the most recent individual patient analysis of eight trials reported that infants who were started with an initial FiO_2_ of < 0.3 had significantly lower 5 min SpO_2_ values compared to infants started with an initial FiO_2_ of ≥ 0.6 [35]. Furthermore, infants initially resuscitated with initial FiO_2_ of 0.21–0.3 compared to initial FiO_2_ of ≥ 0.6 had an increased risk of major intraventricular hemorrhage and a five times higher risk of death [35]. Similarly, if preterm infants resuscitated with initial low FiO_2_ do not reach SpO_2_ of 80% at 5 min, it was associated with increased risk of major intraventricular hemorrhage and an almost five times higher risk of death [35]. There is equally growing evidence that using lower initial FiO_2_ will lead to lower SpO_2_ levels and bradycardia, which may lead to increased rates of mortality in this vulnerable group of infants [34, 36]. These data provide a warning note for the use of higher vs. lower initial FiO_2_ during delivery room resuscitation. Trials are urgently needed to evaluate the risk of using low and high initial FiO_2_ in preterm infant stabilization at birth [37]. In summary, the “optimal” FiO_2_ used to initiate post birth stabilization of extreme preterm seems to be about balancing the need to quickly achieve target saturations while minimizing the oxidative stress of “too much” oxygen.

## Objectives {7}

### Primary research question

The trial is based on the following Population (P), Intervention (I), Comparison (C), Outcome (O), Timeline (T) format: (*P*) in preterm infants born at 23^0^–28^6^ weeks’ gestation, (*I*) does initiating resuscitation with an initial FiO_2_ of 0.6 (*C*) compared to initiating with an initial FiO_2_ of 0.3 (*O*) increase or decrease the incidence of mortality or the presence of major neurodevelopmental outcomes as defined: (i) cerebral palsy with an inability to walk unassisted; (ii) major developmental delay involving cognition or speech, (iii) visual (cannot fixate/legally blind, or corrected acuity < 6/60 in both eyes), or hearing impairment (requiring a hearing aid or cochlear implants) (*T*) at 18–24 months corrected age?

### Secondary objectives

In preterm infants born at 23^0^–28^6^ weeks of gestation, does initiating resuscitation with a higher initial FiO_2_ of 0.6 compared to a lower initial FiO_2_ of 0.3 increase or decrease the components of the primary outcome (death, cerebral palsy, major developmental delay involving cognition, speech, visual, or hearing impairment) in addition to neonatal morbidities (severe brain injury, bronchopulmonary dysplasia; and severe retinopathy of prematurity)?

### Trial design {8}

This is an international, multicenter, cluster, cross-over, superiority randomized controlled trial.

## Methods: participants, interventions, outcomes

### Study setting {9}

The study setting is as follows: delivery rooms of tertiary level perinatal centers in Canada, Ireland, and Spain. All recruiting sites have been entered into ClinicalTrials.gov (https://clinicaltrials.gov/study/NCT03825835) and attached as Appendix.

### Eligibility criteria {10}

Inclusion criteria (all must be satisfied):

- Born between 230/7 and 286/7 weeks’ gestation.
- To receive full resuscitation, i.e., no parental request or pre-determined decision to forego resuscitation.
- No known major congenital or chromosomal malformation.
- Informed parental consent.

### Exclusion criteria

- Infants who are born outside of study center and transported to center after delivery.
- Parental refusal or could not be approached for consent.

### Consent or assent: who will take informed consent? {26a}

Informed parental/guardian consent will be obtained after the study intervention for ongoing data collection and follow-up research staff trained to obtain consent for the trial as per Tri-Council Policy Statement: Ethical Conduct for Research Involving Humans guidelines for research in “Individual Medical Emergencies” [38].

### Consent or assent: ancillary studies {26b}

Participants will be asked if they agree to use of their data should they choose to withdraw from the trial. Participants will also be asked for permission for the research team to share relevant data with people from the universities taking part in the research or from regulatory authorities, where relevant. This trial does not involve collecting biological specimens for storage.

## Interventions

### Choice of comparators {6b}

Over the last 10 years, recommendations regarding the ideal level of oxygen for resuscitation in preterm infants have changed from 100%, down to low levels of oxygen (< 30%), up to moderate concentration (30–65%) [3, 18, 20]. The most recent individual patient analysis of eight trials by Oei et al. reported that infants who were started in < 30% oxygen had significant lower 5 min SpO_2_ values compared to infants started with ≥ 60% oxygen, which was associated with increased risk of major intraventricular hemorrhage and a five times higher risk of death [34]. The authors emphasized that trials are urgently needed to evaluate the risk of using low and higher oxygen concentration in preterm infant stabilization at birth. In addition, in 2010, SpO_2_ targeting was recommended as standard of care, and this contributed to a change in clinical practice as clinicians were more likely and comfortable to start resuscitation at either 21% or titrated levels of oxygen such as 30–40% [18]. When the guidelines were again revised in 2015, the committee acknowledged that a critical knowledge gap continued to exist for the resuscitation of the preterm infants < 37 weeks [20], highlighting the need to provide more concrete guidelines.

This leaves clinicians in a challenging position. Despite the advances that have been achieved in perinatal and neonatal care, neonates are still vulnerable to the consequences of the oxidative effects from hyperoxia as well as the deleterious effects from hypoxia. A large multi-center international trial of sufficient sample size that is powered to look at safety outcomes such as mortality and adverse neurodevelopmental outcomes is required to provide the necessary evidence to guide clinical practice with confidence. The trial will be using 30% and 60% as the two starting oxygen concentrations, both of which are within the parameters of current recommendations and practice.

## Intervention description {11a}

### Setting

All participating centers will change their local hospital policy to either an initial FiO_2_ of 0.3 or 0.6 as per randomization for the first cohort of infants. To enroll the next cohort, participating centers will change their local hospital policy to the other arm with initial FiO_2_ of 0.3 or 0.6.

### Treatment arms

#### ***Initial FiO***_***2***_*** of 0.3 arm***

**Fig. 1:**
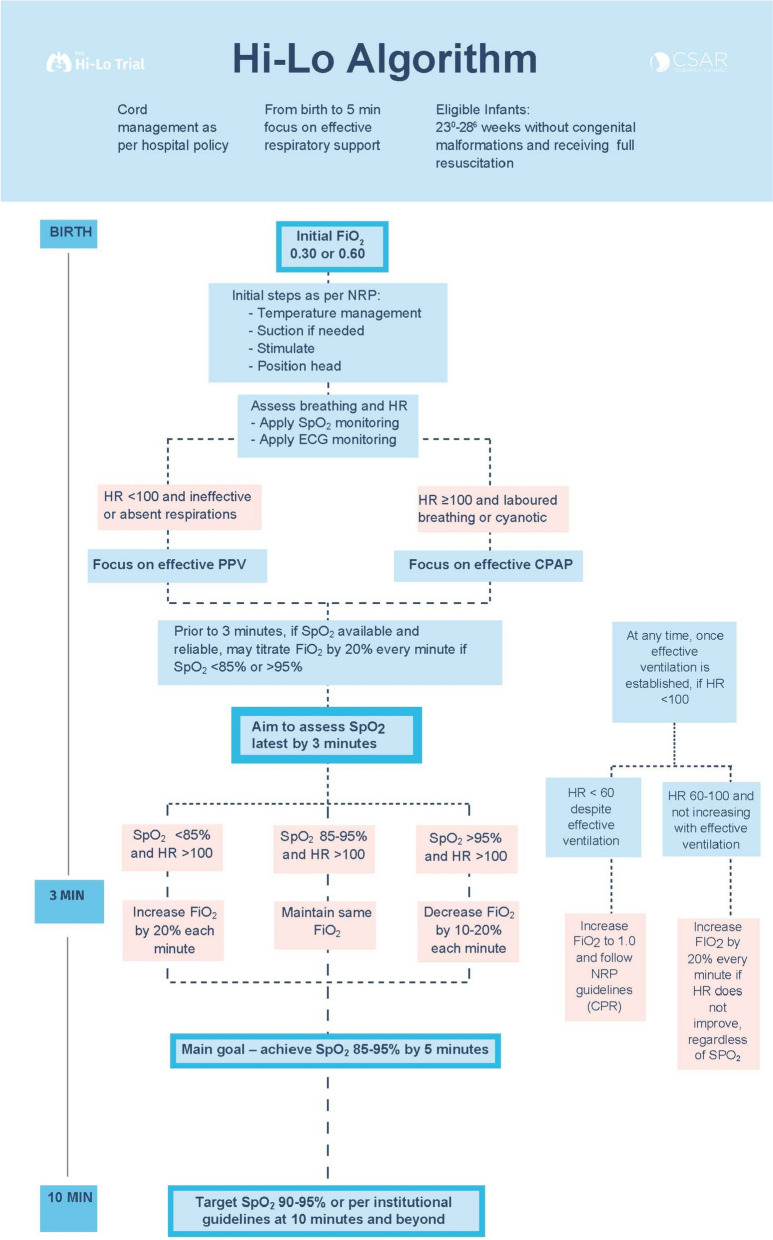
Hi-Lo algorithm

Infants randomized to this arm will receive initial FiO_2_ of 0.3 oxygen during the first 3 min of life. Focus will be on providing effective ventilation. During this time, if SpO_2_ is available and reliable, oxygen may be increased by 20% every minute if an SpO_2_ ≥ 85% is unlikely to be achieved by 5 min. Once a reliable SpO_2_ reading is available, or at the latest at 3 min of age, the clinical team will assess SpO_2_. If SpO_2_ is < 85%, oxygen should be increased by 20% every 60 s to achieve SpO_2_ ≥ 85% at 5 min of age. If SpO_2_ is > 95% at or before 5 min of age, oxygen should be decreased by 10–20% every 60 s to maintain SpO_2_ of ≥ 85%. At 10 min of age and beyond, aim for SpO_2_ of 90–95% or as per institutional guidelines. If, at any time during the resuscitation, the heart rate (HR) is 60–100 and not increasing with effective ventilation, oxygen will be increased by 20% every minute, regardless of SpO_2_ (Fig. 1).

#### ***Initial FiO***_***2***_*** of 0.6 arm***

Infants randomized to this arm will receive initial FiO_2_ of 0.6 during the first 3 min of life. Focus will be on providing effective ventilation. During this time, if SpO_2_ is available and reliable, oxygen may be increased by 20% every minute if an SpO_2_ ≥ 85% is unlikely to be achieved by 5 min. Once a reliable SpO_2_ reading is available, or at the latest at 3 min of age, the clinical team will assess SpO_2_. If SpO_2_ is < 85%, oxygen should be increased by 20% every 60 s to achieve SpO_2_ ≥ 85% at 5 min of age. If SpO_2_ is > 95% at or before 5 min of age, oxygen should be decreased by 10–20% every 60 s to maintain SpO_2_ of ≥ 85%. At 10 min of age and beyond, aim for SpO_2_ of 90–95% or as per institutional guidelines. If, at any time during the resuscitation, the HR is 60–100 and not increasing with effective ventilation, oxygen will be increased by 20% every min, regardless of SpO_2_ (Fig. 1).

### Duration of treatment period

The study intervention duration will be the first 10 min after birth.

### Criteria for discontinuing or modifying allocated interventions {11b}

If HR < 60 at any time despite 30 s of effective ventilation, oxygen will be increased to FiO_2_ 1.0, and resuscitation guidelines will be followed (including chest compressions and epinephrine) (Fig. 1).

### Strategies to improve adherence to interventions {11c}

These include a site initiation visit and training logs, followed by continuing engagement with each site during zoom investigator meeting, annual in-person trail meeting, and monthly trial newsletters.

### Relevant concomitant care permitted or prohibited during the trial {11d}

The intubation of an infant for the sole purpose of prophylactic surfactant administration in a spontaneously breathing infant reaching target saturations will not be allowed in the first 10 min after birth. Other than attempting to achieve the 5-min SpO_2_ targets, all delivery room interventions will follow the center’s local hospital policy (standard hospital practice guideline) and the current neonatal resuscitation guidelines [3, 4].

### Provisions for post-trial care {30}

Care during the primary hospitalization and after post discharge will adhere to local practice guidelines.

## Outcomes {12}

### Primary outcome

The primary outcome will be a composite outcome of all-cause mortality or the presence of a major neurodevelopmental outcome between 18 and 24 months corrected age defined as any one of the following: (i) cerebral palsy with an inability to walk unassisted; (ii) major developmental delay involving cognition or language; or (iii) visual (cannot fixate/legally blind, or corrected acuity < 6/60 in both eyes) or hearing impairment (requiring a hearing aid or cochlear implants).

Between 18 and 24 months corrected age, the infant will be assessed for cerebral palsy, developmental delays, vision, and hearing impairments. An infant who has been diagnosed as having cerebral palsy and assigned a Gross Motor Functional Classification Score (GMFCS) of 3 to 5 will be identified as having an inability to walk unassisted. Infants will be assessed using the Bayley Scales of Infant Development-4th edition (Bayley-IV) to determine major developmental delays related to cognition and speech. A cognitive or language composite score < 70 will be defined as a major delay. Any death following their first discharge home but before the scheduled 18–24-month follow-up visit will also be included in the primary outcome. The Ages & Stages Questionnaires®, 3rd Edition (ASQ-3™, by Squires & Bricker, https://agesandstages.com/products-pricing/asq3) will be an alternative to assess neurodevelopmental outcomes if a child cannot be tested with the Bayley-IV (e.g., pandemic, travel restrictions) [39]. In those cases, the ASQ-3 will be used as an alternative, and sections contributing to cognition and language will be used to determine the level of delay. The ASQ-3 has a similar predicative values and validity to Bayley-III test [40]. Furthermore, to mitigate the problem of higher-than-expected rate of missing values for the primary outcome, the HiLo trial will publish the results of in-hospital mortality (secondary outcome) and severe brain injury on cranial ultrasound (secondary outcome) before the primary composite outcome of all-cause mortality or the presence of a major neurodevelopmental outcome. This will assure a close to 100% ascertainment and prevent the HiLo trial from falling below 100% of achieved sample size, to avoid that trial results become progressively more fragile, and a random or non-random imbalance in the primary outcome among participants with missing values could erase an apparently statistically significant result [41–43].

### Secondary outcomes

Secondary outcomes include the following:

1. Individual components of the composite primary outcomes
2. All-cause in-hospital mortality
3. Severe brain injury on cranial ultrasound: severe grade 3 and 4 intraventricular or intraparenchymal hemorrhage according to Papile [44], periventricular leukomalacia, or ventriculomegaly based on neuroimaging studies (timing and frequency of imaging based on local site practices)
4. Bronchopulmonary dysplasia at 36 weeks corrected age and at 40 weeks corrected age, defined as receiving any supplemental oxygen or any form of respiratory support (including invasive mechanical ventilation, non-invasive ventilation with continuous positive airway pressure, nasal intermittent positive pressure ventilation, or high-flow nasal canula)
5. Severe retinopathy of prematurity (stage 3 or higher) as defined in the International Classification of ROP and/or ROP treated with laser, cryotherapy, or intraocular injection therapy [45]
6. Necrotizing enterocolitis, Modified Bell’s criteria stage 2 or greater [46]
7. Total duration of mechanical ventilation via an endotracheal tube in days
8. Discharge home on oxygen
9. Duration of any positive pressure respiratory support (invasive mechanical ventilation, non-invasive ventilation with continuous positive airway pressure, nasal intermittent positive pressure ventilation, or non-invasive neural assist ventilation or non-invasive high frequency ventilation, or high-flow nasal canula) in days
10. Duration of supplemental oxygen in days
11. Length of hospital stay in days
12. *Z*-scores for weight, length, head circumference, and body mass index at 36 weeks’ post-menstrual age [47]
13. Differences in oxygen saturation at 3, 5, and 10 min of age
14. Proportions of infants at 3 and 5 min who are breathing spontaneously or receiving mask ventilation
15. Proportion of infants receiving FiO_2_ 1.0 during first 10 min of resuscitation
16. Proportion of infants needing to escalate oxygen concentration beyond allocated intervention arm in first 10 min after birth

## Participant timeline {13}

**Table:** 

	**Enrolment**	**Allocation**	**Post-allocation**		**Post-discharge**
**Time point**	Antenatal	At birth	7–10 days of age	Hospital discharge	24 ± 6 months’ (corrected for prematurity)
**Enrolment**					
Eligibility screen	X	X			
Informed consent			X	X	
Maternal demographic and pregnancy data			X		
Randomization data		X			
Baseline infant data			X	X	
**Interventions**					
Intervention		X			
**Outcome assessments**					
Safety assessment in-hospital mortality (part of primary outcome)		X	X	X	X
Safety assessment intraventricular hemorrhage			X		
Completion of admission data				X	
**Primary outcome: neurodevelopmental assessment at 18–24 months corrected age**					X

### Sample size {14}

A sample size of 1200 (600 per group, adjusted for 10% loss of follow-up) infants has been determined based on: baseline outcome rate of 35% (18% mortality and 17% abnormal neurodevelopmental outcomes) [48], RRR of 25%, type I error of 5%, power of 80%, within-cluster within-period ICC of 0.034, within-cluster between-period ICC of 0.029, cluster size in each arm of 10–80 (unequal cluster period but equal cluster size), and loss to follow-up rate of ~ 10%. ICCs were derived from existing data from the Canadian Neonatal Network sites [48]. With these assumptions, we will need up to 20 centers (clusters) to recruit 20–80 patients per arm (20–160/center) for a total of 1200 neonates at all sites.

### Recruitment {15}

All centers were carefully selected as they have previously participated in large randomized trials and have the documented capability of enrolling the required number of infants. All participating centers will change their local hospital policy to either 30 or 60% oxygen as per randomization for the first cohort of infants. As units will switch their policy of initial oxygen concentration for the study period, it is very unlikely that non-compliance will be an issue; however, we will monitor and record carefully. This approach allows all infants born at each center, being automatically included in the trial. The consent approach for the HiLo trial will be unambiguous and acceptable for all participating centers by obtaining individual consent after birth for data inclusion in the trial and follow-up, which will strengths the number being recruited.

The number of infants being recruited will vary between 10 and 70/year depending in the size of the center. The overall recruitment period is a max of 24 months per center; however, if a center reaches their target for the first arm prior of 12 months, the center will switch to the 2nd arm. We estimate ~ 1120 infants being recruited in Canada, ~ 60 infants in Ireland, and ~ 120 infants in Spain.

## Assignment of interventions

### Allocation

#### Sequence generation {16a}

The randomization schedule (as to what will be the first arm a site will be randomized to) is provided by the Biostatics unit, at the Women and Children’s Health Research Institute (WCHRI), University of Alberta, Edmonton, Canada. Before the start of the trial, the statistician will use computer-generated random numbers to prepare the allocation sequence for all participating centers by producing the codes and allocation table, which then will be validated by an independent statistician. Each site will be allocated to either 30% in the first period and 60% in the second period or 60% in the first period and 30% in the second period with 1:1 ratio.

### Concealment mechanism {16b}

The allocation sequence for all participating sites is password protected and the password is only known to the independent statistician, the principal investigator (GMS), and the trial coordinators. Study site allocation was only communicated with each site separately after ethics approval obtained, contracts signed, and study launch confirmed.

### Implementation {16c}

Before the start of the trial, the statistician will use computer-generated random numbers to prepare the allocation sequence for all participating centers by producing the codes and allocation table, which then will be validated by an independent statistician. Clinical staff attending neonatal deliveries know the group assignment and will enroll every participant automatically, as the group assignment is also local hospital policy.

## Assignment of interventions: blinding

### Who will be blinded {17a}

Blinding will not be feasible, as each site will be assigned each study intervention arm until enrollment in the first intervention arm is complete and then switch to the second intervention arm. However, the assessors for the neurodevelopmental outcomes at 18–24 months will be blinded.

### Procedure for unblinding if needed {17b}

While each site knows their group allocation for each study period, there is no need to be unblinded for any severe adverse events (SAE). Members of the data safety monitoring board (DSMB) will have access to unblinded treatment allocation to ascertain causality for any SAE or other serious events that may be attributed to trial participation and at pre-specified intervals for interim efficacy and safety analyses.

## Data collection and management

### Plans for assessment and collection of outcomes {18a}

A manual of operation as well as work sheets have been created for training of site personal for data entry. The manual of operation includes all steps of data collection and data entry. All data entering staff have received an online seminar on how to entre data into REDCap. We have included the data collection form as appendix to this protocol. The neurodevelopmental follow-up assessment will be performed by trained psychologists, developmental pediatrician, neonatologists, or neonatal nurse practitioners, which are all trained in administering the Bayley Scales of Infant Development-4th edition (Bayley-IV). The Ages & Stages Questionnaires® will only be used if a child cannot be tested with the Bayley-IV. The ASQ-3 is a parent answered questionnaire and has a similar predicative values and validity to Bayley-III test [40].

### Plans to promote participant retention and complete follow-up {18b}

We are anticipating a ~ 10% loss to follow-up by the 18–24±﻿6 month-visit, and this has been incorporated into the sample size calculation. The centers have participated in several large neonatal trials with a neurodevelopmental follow-up component with a 3% lost-to-follow-up rate. Centers will be asked to record various contact details as allowed by their research ethics committee to facilitate maintaining contact with the infant’s family.

### Data management {19}

The HiLo investigators and research nurses at each site will be responsible for data collection which will be sourced from the paper or electronic medical charts of the mother and infant. Data will be entered into an electronic database (REDCap™, Vanderbilt University) that will be designed and managed through the University of Alberta. REDCap is a secure web application for building and managing online surveys and databases, designed to support data capture for research studies [49, 50].

### Confidentiality {27}

Participant data will be subject to data protection and privacy laws. All data will be securely stored, electronic records will only be accessible by a password known to the research team, and all data will be de-identified. Anonymity will be preserved in all scientific publications and presentations.

### Biological specimens {33}

There will be no biological specimens collected.

## Statistical methods

### Statistical methods for primary and secondary outcomes {20a}

A detailed statistical analysis plan will be finalized and submitted for publication prior to database lock. Data handling, verification, and analysis for the HiLo trial will be performed by WCHRI. Statistical analysis will follow standard methods for randomized trials, and reporting of findings will be performed in accordance with CONSORT guidelines extension for cluster trials [51].

The primary analysis will be conducted using an “intention-to-treat” approach. For the primary outcome, generalized linear mixed model with binary outcome and maximum likelihood estimate will be used to evaluate the effect of an oxygen concentration on the primary outcome. To account for cluster crossover design of the study, effects of centers (clusters) and a period (oxygen concentration) within center will be considered random, and effects of the intervention (oxygen concentration) will be entered as a fixed effect. This hierarchical model allows for the correlation of patients within periods and within clusters. The model will be adjusted for gestational age and if the infant required mask ventilation as potential confounding variables. To account for multiple births when more than one infant is enrolled in the study, clustering within mothers will be entered as a random effect in the model and this model will be tested for a better fit.

Similar generalized linear mixed models will be performed to evaluate the effect of group on secondary outcomes. If convergence problems arise, different fitting techniques will be used depending on the nature of the problem. For example, different approximation methods (e.g., maximum likelihood or pseudo-likelihood methods) will be tried; different types of covariance structures will be used. In addition, in case of singularity of variance–covariance matrix, models with fewer random effects will be created if the fit is adequate.

Analysis of secondary outcomes will use generalized linear mixed models or descriptive analysis. Summary statistics will be presented for baseline and clinical characteristics: continuous data by mean, two-sided 95% CI of the mean, standard deviation, median, interquartile range (first and third quartiles), minimum and maximum. Categorical data will be presented by absolute and relative frequencies.

### Interim safety analyses {21b}

The DSMB will conduct three interim safety analyses throughout the trial to assess in-hospital mortality after 240 (20%), 400 (33%), and 800 (66%) infants recruited.

### Methods for additional analyses (e.g., subgroup analyses) {20b}

For the primary outcome, the following subgroups will be analyzed: (i) gestational age 23^+0^–25^+6^ vs. 26^+0^–28^+6^; (ii) infants supported with CPAP vs. received mask ventilation [52]; (iii) female vs. male [53, 54].

### Analysis population and missing data {20c}

A sensitivity analysis will be performed to examine the effect of missing values in primary and secondary outcome variables. Multiple imputation will be used for missing data; a very low number of missing values are expected due to study design. To mitigate the problem of higher-than-expected rate of missing values for the primary outcome, the HiLo trial will publish the results of in-hospital mortality (secondary outcome) and severe brain injury on cranial ultrasound (secondary outcome) before the primary composite outcome of all-cause mortality or the presence of a major neurodevelopmental outcome. This will assure a close to 100% ascertainment and prevent the HiLo trial from falling below 100% of achieved sample size, to avoid that trial results become progressively more fragile, and a random or non-random imbalance in the primary outcome among participants with missing values could erase an apparently statistically significant result [41–43].

### Plans to give access to the full protocol, participant level-data and statistical code {31c}

There is full public access to clinical trial registration (NCT03825835; www.clinicaltrials.gov); information is at the Hilo trial website (https://www.hilotrial.org and https://www.research4babies.org/hilo). This study protocol and the statistical analysis plan will be available and submitted for publication.

The complete de-identified HiLo trial dataset collected for this analysis will be available 6 months after publication of the primary outcome. The HiLo trial dataset will be also integrated into the prospective PROspective Meta-analysis Of Trials of Initial Oxygen in preterm Newborns (PROMOTION) [55] and an updated version of the retrospective NETwork Meta-analysis Of Trials of Initial Oxygen in preterm Newborns (NETMOTION) [56]. This study protocol and the statistical analysis plan will also be available and will have been submitted for publication in a journal. An application to obtain the data may be made by emailing georg.schmoelzer@me.com. The final decision to share data will be made by the HiLo trial steering committee.

## Oversight and monitoring

### Composition of the coordinating center and trial steering committee {5d}

The trial management team is based at the Royal Alexandra Hospital, Edmonton, Canada, which includes the principal investigators (GMS) and the trial coordinators (Caroline Fray and Barb Kamstra) and meets weekly. Fabiana Bacchini is the executive director of the Canadian Premature Babies Foundation and has been involved in the trial design.

### Trial steering committee

The trial steering committee detailed below meets approximately quarterly, chaired by GMS.

**Table:** 

Prof Georg M. Schmölzer (Nominated Principal Investigator)	^1^Centre for the Studies of Asphyxia and Resuscitation, Edmonton, Canada University of Alberta, Edmonton, Canada
Prof Prakesh Shah (Principal Investigator)	Mount Sinai Hospital and University of Toronto, Toronto, Canada
Prof Elizabeth V. Asztalos (Principal Investigator)	University of Toronto, Toronto, Canada
Prof William Tarnow-Mordi (Principal Investigator)	University of Sydney, Sydney, Australia
Prof Neil N. Finer (Principal Investigator)	School of Medicine, University of California, and Sharp Mary Birch Hospital for Women and Newborns
Prof Max Vento (Principal Investigator)	University of Valencia, Valencia, Spain
Dr. Maryna Yaskina (Senior Biostatistician)	Women and Children’s Health Research Institute, University of Alberta, Edmonton, Canada
Barb Kamstra (Trial coordinator)	Royal Alexandra Hospital, Edmonton, Canada
Caroline Fray (Trial coordinator)	Royal Alexandra Hospital, Edmonton, Canada
Caroline Fray (Trial coordinator)	Royal Alexandra Hospital, Edmonton, Canada

### Composition of the data monitoring committee, its role, and reporting structure {21a}

The data and safety monitoring board (DSMB) has three independent members (chair, a neonatal clinician, and a biostatistician). The role of the DSMB was outlined in a DSMB Charter finalized prior to the trial commencing. DSMB is responsible to protect and safeguard the interests of all study patients, monitor the overall conduct of the trial, advise the investigators to protect the integrity of the trial, and supervise the conduct and analysis of all interim analyses. The DSMB detailed below meets approximately every 6 months as well as for each interim safety analyses, chaired by GMS.

**Table:** 

Prof Michael Bracken	Chair	New Haven, USA
Prof Christian Poets	Independent Expert	Tuebingen, Germany
A/Prof Kevin Thorpe	Independent Statistician	Toronto, Canada

### Adverse event reporting and harms {22}

Safety reporting from the HiLo trial will follow standards from Health Canada as per section C.05.014 of the Food and Drug Regulations and the Tri-Council Policy Statement: Ethical Conduct for Research Involving Humans [38].

Pre-defined SAE are as follows:

- Death in the delivery room (also a component of the primary outcome)
- Death in the NICU (also a component of the primary outcome)
- Intraventricular hemorrhage grade 3 or higher according to Papile [44]

### Auditing {23}

The Quality Management in Clinical Research (QMCR), University of Alberta, will act as an independent monitor and will perform virtual site data auditing every 3 months to review source documentation. As this trial was classified as regulatory trial according to Health Canada regulations, auditors with Health Canada might also audit the data for adherence.

The ethics committee will meet annually to review conduct, the independent DSMB meets every 6 months to review conduct of study as well as for every interim safety analysis, and the trial steering committee will meet quarterly to review conduct throughout the trial period.

### Plans for communicating important protocol amendments to relevant parties {25}

There have not been any major changes to the trial protocol since the trial began. Minor changes and additions have been submitted for approval to the Health Research Ethics Board (Edmonton, Canada), Health Canada, and all other relevant ethics committees and distributed and communicated to each participating site.

## Dissemination plans

### Trial results {31a}

The results of the trial will be presented at national and international conferences and published in high-impact medical journals. Media and social media opportunities will be sought to communicate the results to the public. We will collaborate with the Canadian Premature Babies Foundation to create lay summary for distribution to all participating families.

## Discussion

Using an initial FiO_2_ of 0.6 may decrease major neurodevelopmental outcomes and improve health benefits in children. The international, cluster randomized HiLo trial is powered to allow the detection of an important difference in the primary outcome of survival free of major neurodevelopmental outcomes at 18–24 months corrected age. The HiLo trial will address gaps in the evidence for the initial oxygen concentration due to its pragmatic and inclusive design, including all extremely preterm infants. Assessment of surviving infants at 18–24 months corrected age (corrected for prematurity) will provide evidence of longer-term efficacy and safety, which is critical for trials in extremely preterm infants.

## Trial status

The current protocol version is 3.5, dated October 4, 2022. Recruitment began in June 2022 at Royal Alexandra Hospital, Edmonton, with additional sites added over time. Recruitment is expected to be completed in early to mid-2026 with results expected in late 2028.

### Supplementary Information

**Supplementary Material 1.**

## Data Availability

The complete de-identified HiLo trial dataset collected for this analysis will be available 6 months after publication of the primary outcome. The HiLo trial dataset will be also integrated into the prospective PROspective Meta-analysis Of Trials of Initial Oxygen in preterm Newborns (PROMOTION) [55] and an updated version of the retrospective NETwork Meta-analysis Of Trials of Initial Oxygen in preterm Newborns (NETMOTION) [56]. This study protocol and the statistical analysis plan will also be available and will have been submitted for publication in a journal. An application to obtain the data may be made by emailing georg.schmoelzer@me.com. The final decision to share data will be made by the HiLo trial steering committee.

## Abbreviations

BPD: Bronchopulmonary dysplasia
CI: Confidence interval
ELBW: Extremely low birth weight
FiO_2_: Fraction of inspired oxygen
HR: Heart rate
RR: Risk ratio
SAE: Severe adverse event
SpO_2_: Oxygen saturation
