# Supplementary material for: Does the use of higher versus lower oxygen concentration improve neurodevelopmental outcomes at 18–24 months in very low birthweight infants?
Source: Trials. 2024 Apr 4;25:237. doi: 10.1186/s13063-024-08080-2 (PMC10996184; doi:10.1186/s13063-024-08080-2)
Supplement: Supplementary file 1 — Supplementary Material 1. [file 13063_2024_8080_MOESM1_ESM.zip › HILO CRF completeR2.pdf]

PID: __ __ __ __- __ __ __

FULL DATA SET

MATERNAL DATA

1. Presenting antenatal problems: *(select all that apply.)*
   - ☐ Premature rupture of membranes (PROM)
   - ☐ Preterm labour
   - ☐ Pregnancy induced hypertension (PIH)
   - ☐ Antepartum hemorrhage (APH)
   - ☐ Intrauterine growth restriction (IUGR) (<10%ile)
   - ☐ Fetal Distress
   - ☐ Other: If other: ____

2. Presentation:
   - ☐ Cephalic
   - ☐ Breech
   - ☐ Transverse
   - ☐ Other: If other: ____

3. Duration of rupture of membranes (ROM) before birth: __ __ __ __ hours (0 if < 1 hour)
   - ☐ Unknown

4. Chorioamnionitis:
   - ☐ Clinically suspected
   - ☐ Histologically proven
   - ☐ No
   - ☐ Other: If other: ____

5. Antenatal steroids:
   - ☐ None
   - ☐ Complete course (two doses)
     - ☐ **Optimal**: two doses, given within one week prior to delivery, 24 hours between doses
     - ☐ **Clinical**: two doses; <u>one or both</u> doses given more than 7 days prior to delivery, <u>OR</u> date of dose administrations unknown
   - ☐ Incomplete course (one dose)
   - ☐ Missing / unknown

6. Administration of MgSo4:
   - ☐ Yes
   - ☐ No
   - ☐ Missing / unknown

INFANT BIRTH DATA

7. Date of Birth: __ __ / __ __ / __ __ __ __ *(dd/mm/yyyy)*

8. Time of Birth: __ __: __ __ *(hh:mm)*

9. Gestation: __ __ weeks __ days

10. Birth Weight: __ __ __ __ grams

11. Sex: ☐ Male ☐ Female ☐ Ambiguous

12. Multiple Birth: ☐ Yes ☐ No If Yes, birth order: ___ of ___

13. Mode of Delivery:
    - ☐ Spontaneous vaginal
    - ☐ Instrumental vaginal
    - ☐ Cesarean Section (C/S)

14. Apgar Score: a. 1 minute __ __ b. 5 minutes: __ __ c. 10 minutes: __ __
    10 min Apgar not done ☐

15. Heart Rate at 1 minute:
    - ☐ < 60 bpm
    - ☐ 60 – 100 bpm
    - ☐ > 100 bpm

DELIVERY ROOM INTERVENTION

16. Cord Management at Delivery:
    - ☐ Immediate Cord Clamping (< 30 seconds)
    - ☐ Delayed Cord Clamping (≥ 30 seconds)
      - If Delayed Cord Clamping done: __ __ __ seconds
      - Did the infant breathe during Delayed cord clamping: ☐ Yes ☐ No
    - ☐ Cord Milking
      - If Umbilical Cord Milking done: # times cord milked __ __
      - Did the infant breath during Cord Milking: ☐ Yes ☐ No
    - ☐ Ventilation Prior to Cord Clamping

17. Oxygen at start of resuscitation: __ __ __ %
    (Complete Protocol Deviation [PD] form if different from site randomization)

18. Maximal Oxygen use during Resuscitation in first 10 minutes: __ __ __ %

19. Was the HiLo algorithm followed during resuscitation: ☐ Yes ☐ No
(If No Complete Protocol Deviation [PD] form)

20. Non-Invasive Positive Pressure Support: ☐ Yes ☐ No
If Yes, Highest level of non-invasive support:
☐ CPAP only
☐ PPV via mask

21. Intubation: ☐ Yes ☐ No
# of intubation attempts (successful & unsuccessful): __ __ *(missing enter 99)*

22. Chest Compressions: ☐ Yes ☐ No
(If Yes fill out REDCap Adverse Event [AE] form)

23. Epinephrine administered: ☐ Yes ☐ No
If Yes, check off which route *(select all that apply):*
☐ Intravenous (IV)
☐ Endotracheal (ETT)
☐ Intraosseous (IO)

24. Surfactant administered during resuscitation: ☐ Yes ☐ No
If Yes: Date: __ __ / __ __ / __ __ __ __ *(dd/mm/yyyy)* Time: __ __: __ __ *(hh:mm)*
(Complete Protocol Deviation [PD] form if given in the first 10 minutes after delivery)

Complete chart below

**Minutes of Age**	**Heart Rate**	**FiO_2**	**SpO_2**	**Respiratory Support (Circle One)**		
3				CPAP	PPV via Mask	ETT
5				CPAP	PPV via Mask	ETT
10				CPAP	PPV via Mask	ETT

25. Status at the end of resuscitation:
☐ Admit to NICU
☐ Died in the delivery room *(Please fill out REDCap Adverse Event [AE] form AND Complete an SAE report form and send to LCC within 48 hrs of investigator becoming aware of death)*

NICU ADMISSION

26. Temperature post stabilization (within 1 hour of birth): __ __. __ °C ☐ Not done

27. Respiratory status on admission:
    - ☐ Conventional Mechanical Ventilation (CMV)
    - ☐ High Frequency Ventilation (HFV)
    - ☐ Non-Invasive Ventilation (CPAP, including NIPPV or Biphasic)
    - ☐ High Flow Nasal Cannula (HFNC)
    - ☐ Low Flow Nasal Cannula (LFNC)

28. Blood gas done within 3 hours of birth: ☐ Yes ☐ No

    If Yes, Sample type:
    - ☐ Arterial
    - ☐ Venous
    - ☐ Capillary

    If Yes, Enter results:

pH	
pCO_2 (mmHg)	
pO_2 (mmHg)	
HCO_3 (mmol/L)	
Base Excess +/- (mmol/L)	
Lactate (mmol/L)	
Hct (L/L)	
Hgb (g/L)	

29. Glucose done within 1 hour of birth: ☐ Yes ☐ No

    If Yes: __ __.__ mmol/L

COURSE IN NICU

(Complete for entirety of infant's NICU stay up to discharge to home, including transfer NICU's/Special Care Nurseries, if available)

Neuro

30. Results of neuroimaging (Head Ultrasound or MRI): (*check all that apply*)
    - ☐ Neuroimaging not done
    - ☐ Normal
    - ☐ Intraventricular Hemorrhage(IVH)
    - ☐ Extensive Leukomalacia
    - ☐ Periventricular Leukomalacia
    - ☐ Porencephalic Cyst
    - ☐ Ventriculomegaly
    - ☐ Other If other: ____

    If Yes to IVH, select the worst grade:
    - ☐ Grade 1
    - ☐ Grade 2
    - ☐ Grade 3
    - ☐ Grade 4

    (If grade 3 or 4, complete REDCap Adverse Event form AND complete an SAE report form and send to LCC within 48 hrs of investigator becoming aware of neuro-imaging results)

Patent Ductus Arteriosus (PDA)

31. Prophylactic Indomethacin: ☐ Yes ☐ No

32. PDA requiring treatment: ☐ Yes ☐ No

    If Yes,
    - ☐ Medically treated:
      # of courses: ______
    - ☐ Surgically treated

Retinopathy of Prematurity (ROP)

33. ROP:
    - ☐ Yes
    - ☐ No
    - ☐ Died prior to assessment
    - ☐ Unknown post transfer from study site
    - ☐ Not assessed

    If Yes,

    Stage: Right Eye: ☐ 0 ☐ 1 ☐ 2 ☐ 3 ☐ 4 ☐ 5 ☐ unknown / missing
    Left Eye: ☐ 0 ☐ 1 ☐ 2 ☐ 3 ☐ 4 ☐ 5 ☐ unknown / missing

    Plus Disease: Right Eye: ☐ yes ☐ no ☐ missing
    Left Eye: ☐ yes ☐ no ☐ missing

Treated for ROP: ☐ Yes ☐ No ☐ Unknown / Missing

If yes, treatment type:
☐ Laser therapy
☐ Cryotherapy
☐ Monoclonal antibody therapy
☐ Other
If other: ____

Necrotizing Enterocolitis (NEC)

34. NEC – Bell's stage 2 or greater: ☐ Yes ☐ No
If Yes:
☐ Medically treated
☐ Surgically treated

Sepsis

35. Early onset sepsis (<72 hours of age) requiring treatment: ☐ Yes ☐ No
If Yes, Body fluid *(check all that apply):*
☐ Blood
☐ CSF
☐ Urine

36. Late onset sepsis (≥72 hours of age) requiring treatment: ☐ Yes ☐ No
If yes, Body fluid *(check all that apply):*
☐ Blood
☐ CSF
☐ Urine

Respiratory

37. Did infant require mechanical ventilation: ☐ Yes ☐ No
If Yes:
- Duration of Conventional Mechanical Ventilation: __ __ __ days
- Duration of High Frequency Ventilation: __ __ __ days

38. Did infant require inhaled nitric oxide (iNO): ☐ Yes ☐ No
If Yes:
- Duration of iNO: __ __ __ hours

39. Did infant require non-invasive ventilation, pressure support or oxygen therapy: ☐ Yes ☐ No

If Yes:

- Duration of CPAP (including NIPPV or biphasic): __ __ __ days
- Duration of High Flow Nasal Cannula: __ __ __ days
- Duration of Low Flow Nasal Cannula: __ __ __ days

40. Respiratory Complications:

☐ None
☐ Pulmonary hemorrhage
☐ Pulmonary hypertension
☐ Pulmonary interstitial emphysema
☐ Other If other: ____

41. Was there a radiologic diagnosis of pulmonary air leak including pneumothorax, pneumomediastinum or pulmonary interstitial emphysema (PIE) during the NICU stay:

☐ Yes ☐ No

If Yes, was it diagnosed within 48 hours of delivery? ☐ Yes ☐ No

(If yes complete REDCap Adverse Event form)

42. Postnatal steroids for treatment of BPD: ☐ Yes ☐ No

If Yes:

- 1st course start date: __ __/__ __/__ __ __ __ 1st course end date: __ __/ __ __/ __ __ __ __
- 2nd course start date: __ __/__ __/__ __ __ __ 2nd course end date: __ __/ __ __/ __ __ __ __
- 3rd course start date: __ __/__ __/__ __ __ __ 3rd course end date: __ __/ __ __/ __ __ __ __

Day 28

43. Infant status at 28 days:

☐ Died
☐ Remains in NICU/Special Care/Level II Nursery
☐ Unknown – transferred, data unavailable

44. Respiratory support at 28 days:

☐ None ☐ HFNC > 2L/min
☐ Mechanical Ventilation ☐ NC ≤ 2L/min
☐ NIPPV ☐ Missing / Unknown
☐ CPAP

45. Oxygen therapy at 28 days: ☐ Yes ☐ No ☐ Unknown

If Yes:

FiO_2 (range 0.22-1.0) or Flow rate (if LFNC): _________________

WEEK 36 / DISCHARGE

46. Infant status at 36 weeks:
    - ☐ Died
    - ☐ Remains in NICU/Special Care/Level II Nursery
    - ☐ Discharged home
    - ☐ Unknown – transferred, data unavailable

47. Head circumference at 36 weeks (or prior to discharge home): __ __.__ cm ☐ Missing / Unknown

48. Weight at 36 weeks (or prior to discharge home): __ __ __ __ grams ☐ Missing / Unknown

49. Respiratory support at 36 weeks (or prior to discharge home):
    - ☐ None
    - ☐ Mechanical Ventilation
    - ☐ NIPPV
    - ☐ CPAP
    - ☐ HFNC > 2L/min
    - ☐ NC ≤ 2L/min
    - ☐ Missing / Unknown

50. Oxygen therapy at 36 weeks (or prior to discharge home): ☐ Yes ☐ No ☐ Unknown
    If Yes:
    FiO_2 (range 0.22-1.0) or Flow rate (if LFNC): ________________

WEEK 40 / DISCHARGE

51. Infant status at 40 weeks:
    - ☐ Died
    - ☐ Remains in NICU/Special Care/Level II Nursery
    - ☐ Discharged home
    - ☐ Unknown – transferred, data unavailable

52. Respiratory support at 40 weeks (or prior to discharge home):
    - ☐ None
    - ☐ Mechanical Ventilation
    - ☐ NIPPV
    - ☐ CPAP
    - ☐ HFNC > 2L/min
    - ☐ NC ≤ 2L/min
    - ☐ Missing / Unknown

53. Oxygen therapy at 40 weeks (or prior to discharge home): ☐ Yes ☐ No ☐ Unknown
    If Yes:
    FiO_2 (range 0.22-1.0) or Flow rate (if LFNC): ________________

CONGENITAL ABNORMALITIES

1. Were there any congenital or chromosomal abnormalities identified during the NICU stay: ☐ Yes ☐ No

   If Yes, describe: ____

CO-ENROLLMENT

2. Was the infant co-enrolled in other research trials: ☐ Yes ☐ No

   If Yes, list co-enrolled trials: ____
   ____
   ____

Completed By: ___________________________ Date: __ __ / __ __ / __ __ __ __ *(dd/mm/yyyy)*

END OF NICU STAY

Disposition at end of NICU/Special Care/Level II Nursery stay:

1. ☐ Discharged home:
2. ☐ Transferred to pediatric ward,
3. ☐ Remains in NICU at 48 weeks PMA
4. ☐ Transfer out of study center, unable to obtain further data

For above 4 options: Date of disposition: __ __ / __ __ / __ __ (dd/mm/yyyy)
Last weight obtained: _______________ grams
On oxygen therapy at disposition: ☐ Yes ☐ No

5. ☐ Died prior to NICU discharge: Date of death: __ __ / __ __ / __ __ (dd/mm/yyyy)

If died prior to discharge complete an Adverse Event form in REDCap AND complete an SAE report form. Send SAE report form to LCC within 48 hours of investigator becoming aware of death.

Primary cause of death (choose one)

☐ Pulmonary hypoplasia
☐ Severe RDS
☐ CLD
☐ Pneumonia
☐ Grade 3 or 4 IVH
☐ Other: ____
☐ Meningitis
☐ Septicemia
☐ NEC
☐ Sudden unexplained death

Secondary cause of death (choose all that apply)

☐ Pulmonary hypoplasia
☐ Extreme prematurity
☐ Severe RDS
☐ CLD
☐ Pneumonia
☐ Other: ____
☐ Grade 3 or 4 IVH
☐ Meningitis
☐ Septicemia
☐ NEC
☐ Sudden unexplained death

Completed By: ____________________________ Date: __ __ / __ __ / __ __ __ __ *(dd/mm/yyyy)*

END OF STUDY FORM

Complete this form once all data for this patient has been collected and entered into REDCap, including Adverse Events and Protocol Deviations (if applicable) to allow for data locking.

1. End of study status:
   - ☐ Minimal data set only (unable to obtain parental consent)
   - ☐ Enrolled (consented) & follow-up complete
   - ☐ Enrolled (consented) & lost to follow-up
   - ☐ Enrolled (consented) & parents withdrew consent
   - ☐ Enrolled (consented) & died before follow-up

2. Study end date: __ __ / __ __ / __ __ __ __ *(dd/mm/yyyy)*

Completed By: ______________________________ Date: __ __ / __ __ / __ __ __ __ *(dd/mm/yyyy)*

PID: __ __ __ __- __ __ __

THE Hi-Lo Trial

MINIMAL DATA SET

INFANT BIRTH DATA

1. Gestation: __ __ weeks __ __ days

2. Birth Weight: __ __ __ __ grams

3. Sex: ☐ Male ☐ Female ☐ Ambiguous

4. Multiple Birth: ☐ Yes ☐ No
   If Yes, birth order: ___ of ___

5. Mode of Delivery:
   ☐ Spontaneous vaginal
   ☐ Instrumental vaginal
   ☐ Cesarean Section (C/S)

6. Apgar Score: a. 1 minute __ __ b. 5 minutes: __ __ c. 10 minutes: __ __
   10 min Apgar not done ☐

7. Heart Rate at 1 minute:
   ☐ < 60 bpm
   ☐ 60 – 100 bpm
   ☐ > 100 bpm

DELIVERY ROOM INTERVENTIONS

8. Cord Management at Delivery:
   ☐ Immediate Cord Clamping (< 30 seconds)
   ☐ Delayed Cord Clamping (≥ 30 seconds)
      If Delayed Cord Clamping done: __ __ __ seconds
      Did the infant breathe during Delayed cord clamping: ☐ Yes ☐ No
   ☐ Cord Milking
      If Umbilical Cord Milking done: # times cord milked __ __
      Did the infant breath during Cord Milking: ☐ Yes ☐ No
   ☐ Ventilation Prior to Cord Clamping

9. Oxygen at start of resuscitation: __ __ __ %
   (Complete Protocol Deviation [PD] form if different from site randomization)

10. Maximal Oxygen use during Resuscitation in first 10 minutes: __ __ __ %

11. Was the HiLo algorithm followed during resuscitation: ☐ Yes ☐ No
(If No Complete Protocol Deviation [PD] form)

12. Non-Invasive Positive Pressure Support: ☐ Yes ☐ No
If yes, Highest level of non-invasive support
☐ CPAP only
☐ PPV via mask

13. Intubation: ☐ Yes ☐ No
\# of intubation attempts(successful & unsuccessful): __ __ *(missing enter 99)*

14. Chest Compressions: ☐ Yes ☐ No
(If Yes fill out REDCap Adverse Event [AE] form)

15. Complete chart below:

Minutes of Age	Heart Rate	FiO_2	SpO_2	Respiratory Support (Circle One)		
3				CPAP	PPV via Mask	ETT
5				CPAP	PPV via Mask	ETT
10				CPAP	PPV via Mask	ETT

16. Status at the end of resuscitation:
☐ Admit to NICU
☐ Died in the delivery room *(Complete a REDCap Adverse Event [AE] form AND Complete an SAE report form and send to LCC within 48 hrs of investigator becoming aware of death)*

COURSE IN NICU

Neuro

17. Results of neuroimaging (Head Ultrasound or MRI): (*check all that apply*)

☐ Neuroimaging not done
☐ Normal
☐ Intraventricular Hemorrhage (IVH)
☐ Extensive Leukomalacia
☐ Periventricular Leukomalacia
☐ Porencephalic Cyst
☐ Ventriculomegaly
☐ Other

If other: ____

If IVH, select worst grade:
☐ Grade 1
☐ Grade 2
☐ Grade 3
☐ Grade 4

(If grade 3 or 4, complete REDCap Adverse Event form AND complete an SAE report form and send to LCC within 48 hrs of investigator becoming aware of neuro-imaging results)

Respiratory

18. Was there a radiologic diagnosis of pulmonary air leak including pneumothorax, pneumomediastinum or pulmonary interstitial emphysema (PIE) during the NICU stay:

☐ Yes ☐ No

If Yes, was it diagnosed within 48 hours of delivery ☐ Yes ☐ No

(If yes complete REDCap Adverse Event form)

Completed By: _____________________________ Date: __ __ / __ __ / __ __ __ __ *(dd/mm/yyyy)*

THE Hi-Lo Trial

END OF NICU STAY

Disposition at end of NICU/Special Care/Level II Nursery stay:

1. ☐ Discharged home:
2. ☐ Transferred to pediatric ward,
3. ☐ Remains in NICU at 48 weeks PMA
4. ☐ Transfer out of study center, unable to obtain further data

For above 4 options: Date of disposition: __ __ / __ __ / __ __ (dd/mm/yyyy)
Last weight obtained: _______________ grams
On oxygen therapy at disposition: ☐ Yes ☐ No

5. ☐ Died prior to NICU discharge: Date of death: __ __ / __ __ / __ __ (dd/mm/yyyy)

If died prior to discharge, complete an Adverse Event form in REDCap AND complete an SAE report form. Send SAE report form to LCC within 48 hrs of investigator becoming aware of death)

Primary cause of death (choose one)

☐ Pulmonary hypoplasia
☐ Severe RDS
☐ CLD
☐ Pneumonia
☐ Grade 3 or 4 IVH
☐ Other: ____
☐ Meningitis
☐ Septicemia
☐ NEC
☐ Sudden unexplained death

Secondary cause of death (choose all that apply)

☐ Pulmonary hypoplasia
☐ Extreme prematurity
☐ Severe RDS
☐ CLD
☐ Pneumonia
☐ Other: ____
☐ Grade 3 or 4 IVH
☐ Meningitis
☐ Septicemia
☐ NEC
☐ Sudden unexplained death

Completed By: ____________________________ Date: __ __ / __ __ / __ __ __ __ *(dd/mm/yyyy)*

END OF STUDY FORM

Complete this form once all data for this patient has been collected and entered into REDCap, including Adverse Events and Protocol Deviations (if applicable) to allow for data locking.

1. End of study status:
   - ☐ Minimal data set only (unable to obtain parental consent)
   - ☐ Enrolled (consented) & follow-up complete
   - ☐ Enrolled (consented) & lost to follow-up
   - ☐ Enrolled (consented) & parents withdrew consent
   - ☐ Enrolled (consented) & died before follow-up

2. Study end date: __ __ / __ __ / __ __ __ __ *(dd/mm/yyyy)*

Completed By: ____________________________ Date: __ __ / __ __ / __ __ __ __ *(dd/mm/yyyy)*

LONG-TERM NEURODEVELOPMENTAL OUTCOMES (18-24 MONTHS ADJUSTED AGE)

1. Did the infant have 2 year follow up assessment: ☐ Yes ☐ No

   If Yes, Type of contact: ☐ Clinic Visit ☐ Phone Call Only
   Date assessment completed: __ __ / __ __ / __ __ *(dd/mm/yyyy)*

   If no, state reason:
   ☐ Died after discharge
   ☐ Lost to follow-up
   ☐ Parents declined follow-up
   If died after discharge, last known date alive: __ __ / __ __ / __ __ *(dd/mm/yyyy)*
   If no, fill in reason, skip to end and sign and date

2. Does child have vision impairment: ☐ Yes ☐ No
   If Yes:
   a. Type of vision impairment: ☐ Unilateral ☐ Bilateral
   b. Outcome of vision impairment:
      ☐ Functional with corrective lenses
      ☐ Blind, some functional vision
      ☐ Blind, no useful vision

3. Does child have hearing impairment: ☐ Yes ☐ No
   If Yes:
   a. type of hearing impairment:
      ☐ Unilateral ☐ Bilateral
   b. Outcome of vision impairment:
      ☐ Has some hearing problems but does NOT need a hearing aid
      ☐ Hears well or with only a little difficulty WITH a hearing aid
      ☐ Has severe hearing difficulty with a hearing aid or hearing is not helped with an aid

4. Has this child been diagnosed with cerebral palsy: ☐ Yes ☐ No
   If Yes, is the CP
   ☐ Mild (unsteady walk)
   ☐ Moderate (unable to walk without help)
   ☐ Severe (cannot walk)
   ☐ Missing / Unknown

   Gross Motor Function Classification System (GMFCS) level: ☐ 1 ☐ 2 ☐ 3 ☐ 4 ☐ 5
   ☐ Unknown

5. Does this child have problems with language or speech: ☐ Yes ☐ No

6. Does this child use more than 10 recognizable words: ☐ Yes ☐ No
   (includes signed words)

7. Was the infant admitted to the hospital since discharge:
   ☐ Yes
   ☐ No
   ☐ Missing

   If Yes:
   a. Number of hospital admissions: __ __
   b. Reason for admission(s): ____
   ____
   ____

8. Respiratory Medications since discharge: ☐ Yes ☐ No

   If Yes, list what medications: ______________________________________

9. Does this child have any major medical problems or diagnosis: ☐ Yes ☐ No
   If Yes, please describe: ____

If unable to attend clinic visit/complete BSID IV assessment , complete question #10, otherwise go to question #11

ASQ 3 (Ages and Stages Questionnaires)

10. Assessment complete: ☐ Yes ☐ No

    If No, reason not complete: ____

    If Yes:
    Date ASQ-3 completed: __ __ / __ __ / __ __ *(dd/mm/yyyy)*

    a. Communication total score: ___ ___
    b. Gross motor total score: ___ ___
    c. Fine motor total score: ___ ___
    d. Problem solving total score: ___ ___
    e. Personal-social total score: ___ ___

BSID IV (Bayley Scales of Infant and Toddler Development Edition IV)

11. Assessment complete: ☐ Yes ☐ No

If No, reason not complete: ____

If Yes:
Date BSID IV completed: __ __ / __ __ / __ __ *(dd/mm/yyyy)*

Cognitive
a. Cognitive raw score: __ __ __
b. Cognitive scaled score: __ __ __
c. Cognitive composite score: __ __ __

Language
d. Receptive communication raw score: __ __ __
e. Expressive communication raw score: __ __ __
f. Receptive communication scaled score: __ __ __
g. Expressive communication scaled score: __ __ __
h. Language sum: __ __ __
i. Language composite score: __ __ __

Motor
j. Fine motor raw score: __ __ __
k. Gross motor raw score: __ __ __
l. Fine motor scaled score: __ __ __
m. Gross motor scaled score: __ __ __
n. Motor sum: __ __ __
o. Motor composite score: __ __ __

Social-Emotional
p. Social-emotional raw score: __ __ __
q. Social-emotional scaled score: __ __ __
r. Social-emotional composite score: __ __ __

Completed By: ____________________________ Date: __ __ / __ __ / __ __ __ __ *(dd/mm/yyyy)*

PROTOCOL DEVIATION FORM

1. Did a protocol deviation occur: ☐ Yes ☐ No

   If Yes:
   ☐ Consent procedures *(wrong consent version used; consent form missing; date/signatures missing*)
   ☐ AE reporting *(Not reporting within 48 hrs; not reporting to REB)*
   ☐ Wrong oxygen concentration used at start of resuscitation
   ☐ HiLo delivery algorithm not followed during resuscitation
   ☐ Surfactant given in the first 10 minutes of life
   ☐ Other

2. Description of protocol deviation:
   ____
   ____
   ____
   ____

3. Date deviation occurred: __ __ / __ __ / __ __ __ __ *(dd/mm/yyyy)*

4. Date REB notified (if applicable): __ __ / __ __ / __ __ __ __ *(dd/mm/yyyy)*

Completed By: ______________________________ Date: __ __ / __ __ / __ __ __ __ *(dd/mm/yyyy)*

PID: __ __ __ __- __ __ __

THE Hi-Lo Trial

ADVERSE EVENT FORM

*** Complete a Separate Form for each adverse event*

1. Did an adverse event (AE) occur: ☐ Yes ☐ No

   If Yes:

   a. ☐ Pulmonary air leak in 1st 48 hours
   b. ☐ Cardiac compressions in the delivery room
   c. ☐ Grade III or IV intraventricular hemorrhage
   d. ☐ Any condition leading to death in the delivery room
   e. ☐ Any condition leading to death in the NICU
   f. ☐ Any other event deemed to be and AE by Site Investigator

   For events c, d, & e (Protocol defined SAE's):
   Complete a REDCap Adverse Event [AE] form
   AND
   *Complete a **Serious Adverse Event** report form and send to LCC within 48 hours of investigator becoming aware of event)*

2. Description of AE:

   ____
   ____
   ____
   ____
   ____

3. Reported term for the Adverse Event: ________________________________

   (if Death, please use primary cause)

4. Severity of AE:

   ☐ Mild
   ☐ Moderate
   ☐ Severe
   ☐ Life threatening
   ☐ Death

5. Attribution to HiLo intervention:

   ☐ Unrelated
   ☐ Unlikely
   ☐ Possibly
   ☐ Probably
   ☐ Definitely

6. Was the event serious? ☐ Yes ☐ No

7. Start date: __ __ / __ __ / __ __ __ __ (dd/mm/yyyy) Start time: __ __:__ __ (hh:mm)

8. Stop date: __ __ / __ __ / __ __ __ __ (dd/mm/yyyy) Stop time: __ __: __ __ (hh:mm)

9. Outcome of AE:
   - ☐ Recovered / resolved with no sequelae
   - ☐ Recovering / resolving
   - ☐ Not recovered / unresolved
   - ☐ Recovered / resolved with sequelae
   - ☐ Fatal

Completed By: ______________________________ Date: __ __ / __ __ / __ __ __ __ *(dd/mm/yyyy)*
