# Supplementary material for: Does the use of higher versus lower oxygen concentration improve neurodevelopmental outcomes at 18–24 months in very low birthweight infants?
Source: Trials. 2024 Apr 4;25:237. doi: 10.1186/s13063-024-08080-2 (PMC10996184; doi:10.1186/s13063-024-08080-2)
Supplement: Supplementary file 1 — Supplementary Material 1. [file 13063_2024_8080_MOESM1_ESM.zip › HiLoTrial REDCap CRF Jan-24R2.pdf]

Enrollment

Participant Identification Number (PID #) ______________________

Complete for all inborn infants delivered between 23+0 and 28+6 weeks gestation that received resuscitation at birth

Hospital Randomization ◯ 30% ◯ 60%
((minimal dataset))

Section A: Enrollment

Enrolled? ◯ Yes ◯ No

Why not enrolled
◯ Parents declined
◯ Parents unavailable
◯ Died/discharged prior to talking with parents
◯ Language barrier
◯ Other

If parents declined, no further data collection - does not qualify for minimal data if ineligible or refusal

Other ______________________

Date of consent: ______________________
(dd-mm-yyyy)

Maternal And Infant Data

Infant not eligible or parents declined consent

Consent not obtained - collect minimal dataset only

Section B: Maternal Data

Presenting antenatal problems	☐ Premature Rupture of Membranes (PROM)<br>☐ Preterm labour<br>☐ Pregnancy Induced Hypertension (PIH)<br>☐ Antepartum Hemorrhage (APH)<br>☐ Intrauterine Growth Restriction (IUGR) (< 10%ile)<br>☐ Fetal distress<br>☐ Others<br>(select all that apply)
If other:	________________________
Presentation	◯ Cephalic ◯ Breech<br>◯ Transverse ◯ Other
If other	________________________
Duration of Rupture of Membranes (ROM) before birth number of hours	________________________<br>(hours)
	☐ Duration of ROM unknown
Chorioamnionitis	◯ Clinically suspected<br>◯ Histologically proven<br>◯ No<br>◯ Other
If other:	________________________
Antenatal steroids	◯ None<br>◯ Complete course (2 doses)<br>◯ Incomplete course (1 dose)<br>◯ Missing / unknown
Complete course (2 doses):	◯ Optimal: two doses, given within one week prior to delivery, 24 hours between doses<br>◯ Clinical: two doses; one or both doses given more than 7 days prior to delivery, OR date of dose administrations unknown
Administration of MgSo4:	◯ Yes ◯ No ◯ Missing/unknown

Section C: Infant birth data

Date and Time of Birth

____
((dd-mm-yyyy hh:mm))

Gestation (weeks)

____
(weeks (minimal dataset))

Gestation (days)

____
(days (minimal dataset))

Please check your data entry. Study forms should be completed for all inborn infants delivered between 23+0 and 28+6 weeks gestation who received active resuscitation at birth

Birth weight

____
(grams (minimal dataset))

Sex

○ Male ○ Female ○ Ambiguous
((minimal dataset))

Multiple Birth

○ Yes ○ No
((minimal dataset))

Birth order

____
((minimal dataset))

of:

____
((minimal dataset) Birth order: [inf_birth_order] of [inf_birth_num])

Please check data entry. Birth order must not be greater than the number of infants in the pregnancy.

Mode of Delivery

○ Spontaneous vaginal
○ Instrumental vaginal
○ Cesarean section (C/S)
((minimal dataset))

Apgar Score at 1 Minute

____
((minimal dataset))

Apgar Score at 5 Minutes

____
((minimal dataset))

Apgar Score at 10 Minutes

____
((minimal dataset))

☐ Apgar Score at 10 minutes Not done

Heart Rate at 1 Minute	◯ < 60 bpm ◯ 60-100 bpm<br>◯ >100 bpm<br>((minimal dataset))

Delivery Room Interventions

Infant not eligible or parents declined consent

Consent not obtained - collect minimal dataset only

Section D: Delivery Room interventions

Cord Management at delivery	◯ Immediate Cord Clamping (< 30s)<br>◯ Delayed Cord Clamping (≥ 30s)<br>◯ Cord milking<br>◯ Ventilation prior cord clamping<br>((minimal dataset))
If Delayed Cord Clamping done:	________________________<br>(seconds (minimal dataset))
Did the infant breathe during Delayed Cord Clamping	◯ Yes ◯ No<br>((minimal dataset))
If Umbilical Cord Milking done: # times cord milked	________________________<br>((minimal dataset))
Did the infant breathe during Cord Milking;	◯ Yes ◯ No<br>((minimal dataset))
% Oxygen at start of Resuscitation	________________________<br>(Percent (%) (minimal dataset))
% Oxygen does not match the randomization. Please check your data entry and if correct, enter a protocol deviation.	
% Maximal Oxygen during Resuscitation in first 10 minutes	________________________<br>(Percent (%) (minimal dataset))
% Maximal Oxygen can not be less than the % Oxygen at start of Resuscitation.	
Was the HiLo algorithm followed during resuscitation:	◯ Yes ◯ No
If No Complete Protocol Deviation [PD] form.	
Non-Invasive Positive Pressure Support	◯ Yes ◯ No<br>((minimal dataset))
Highest level of non-invasive support:	◯ CPAP only ◯ PPV via mask<br>((minimal dataset))
Intubation	◯ Yes ◯ No<br>((minimal dataset))

Number of Intubation attempts	__________________________________ (integer (range 0-15, 99=missing)(minimal dataset))
Please check data entry. (range 0-15, 99=missing)	
Chest compressions	◯ Yes ◯ No ((minimal dataset))
If Yes fill out REDCap Adverse Event [AE] form	
Epinephrine administered	◯ Yes ◯ No
Route	☐ Intravenous (IV)<br>☐ Endtotracheal Tube (ETT)<br>☐ intraosseous (IO)<br>(select all that apply)
Surfactant administered in Delivery Room	◯ Yes ◯ No
Date/time	__________________________________ (dd-mm-yyyy hh:mm)
Age Surfactant Given	__________________________________ (minutes)
Complete Protocol Deviation [PD] form if given in the first 10 minutes of life	
3 minutes of age	
Heart Rate	__________________________________ (beats/min(minimal dataset))
	☐ Heart Rate not available ((minimal dataset))
FiO2	__________________________________ (number with 2 decimal places(minimal dataset))
SpO2	__________________________________ (Percent (%)(minimal dataset))
	☐ SpO2 not available ((minimal dataset))
Respiratory Support	◯ CPAP ◯ PPV via mask<br>◯ ETT<br>((minimal dataset))
5 minutes of age	

Heart Rate	______________ (beats/min(minimal dataset))
	☐ Heart Rate not available ((minimal dataset))
FiO2	______________ (number with 2 decimal places(minimal dataset))
SpO2	______________ (Percent (%)(minimal dataset))
	☐ SpO2 not available ((minimal dataset))
Respiratory Support	◯ CPAP ◯ PPV via mask ◯ ETT ((minimal dataset))
10 minutes of age	
Heart Rate	______________ (beats/min(minimal dataset))
	☐ Heart Rate not available ((minimal dataset))
FiO2	______________ (number with 2 decimal places(minimal dataset))
SpO2	______________ (Percent (%)(minimal dataset))
	☐ SpO2 not available ((minimal dataset))
Respiratory Support	◯ CPAP ◯ PPV via mask ◯ ETT ((minimal dataset))
Please fill out REDCap Adverse Event [AE] form AND Complete an SAE report form and send to LCC within 48 hrs of investigator becoming aware of death	
Status at end resuscitation	◯ Admit to NICU ◯ Died in delivery room (complete AE form) ((minimal dataset))

NICU Admission

Infant not eligible or parents declined consent

Consent not obtained - collect minimal dataset only

Section E: NICU Admission

Temperature post stabilization (within 1 hour of birth)	______________ (Degrees Celcius)
	☐ Temp not done
Respiratory status on admission	◯ Conventional Mechanical Ventilation (CMV)<br>◯ High Frequency Ventilation (HFV)<br>◯ Non-invasive ventilation (CPAP including NIPPV or Biphasic)<br>◯ High flow nasal cannula (HFNC)<br>◯ Low flow nasal cannula (LFNC)
Blood gas done within 3 hours of birth	◯ Yes ◯ No
Sample type	◯ Arterial ◯ Venous<br>◯ Capillary
pH<br>☐ pH not available	
pH	______________ (number with 2 decimal places)
pCO2<br>☐ pCO2 not available	
pCO2	______________ (mmHg)
pO2<br>☐ pO2 not available	
pO2	______________ (mmHg)
HCO3<br>☐ HCO3 not available	

HCO3

____
(mmol/L number with 1 decimal place)

Base Excess (BE)

☐ Base Excess (BE) not available

Base Excess (BE) +/-

____
(mmol/L number with 1 decimal place)

Lactate

☐ Lactate not available

Lactate

____
(mmol/L)

Hct

☐ Hct not available

Hct

____
(L/L (number with 2 decimal places))

Hgb

☐ Hgb not available

Hgb

____
(g/L)

Was a glucose done within 1 hour birth? ◯ Yes ◯ No

Glucose

Glucose

____
(mmol/L number with one decimal place)

Section F: Course in NICU (all infants)
Complete for the entirety of the infant's NICU stay up to discharge to home, including transfer NICU's & Special Care Nurseries, if available

Neuro

Results of Neuroimaging (Head Ultrasound or MRI):	☐ Neuroimaging not done<br>☐ Normal<br>☐ Intraventricular Hemorrhage(IVH)<br>☐ Extensive leukomalacia<br>☐ Periventricular leukomalacia<br>☐ Porencephalic cyst<br>☐ Ventriculomegaly<br>☐ Other<br>((minimal dataset))
If Other: (Describe)	__________________________________<br>((minimal dataset))
Worst Grade of IVH:	◯ Grade 1<br>◯ Grade 2<br>◯ Grade 3 (complete AE form)<br>◯ Grade 4 (complete AE form)<br>◯ Missing<br>((minimal dataset))
If grade 3 or 4, complete REDCap Adverse Event form AND complete an SAE report form and send to LCC within 48 hrs of investigator becoming aware of neuro-imaging results	
Patent Ductus Arteriosis (PDA)	
Prophylactic Indomethacin	◯ Yes ◯ No
PDA requiring treatment	◯ Yes ◯ No
Medically treated	◯ Yes ◯ No
Number of courses	__________________________________
Surgically treated	◯ Yes ◯ No
Retinopathy of Prematurity (ROP)	
ROP	◯ Yes<br>◯ No<br>◯ Died prior to assessment<br>◯ Discharged/transferred prior to assessment<br>◯ Unknown post transfer from study site<br>◯ Not assessed
Worst Stage - Right Eye:	◯ 0<br>◯ 1<br>◯ 2<br>◯ 3<br>◯ 4<br>◯ 5<br>◯ Unknown/missing

Worst Stage - Left Eye:	◯ 0<br>◯ 1<br>◯ 2<br>◯ 3<br>◯ 4<br>◯ 5<br>◯ Unknown/missing
Plus disease - Right eye:	◯ Yes ◯ No ◯ Missing
Plus disease - Left eye:	◯ Yes ◯ No ◯ Missing
Treated for ROP	◯ Yes ◯ No ◯ Unknown/missing
Treatment type	☐ Laser therapy<br>☐ Cryotherapy<br>☐ Monoclonal antibody therapy<br>☐ Other
If other:	__________________________
Necrotizing Enterocolitis (NEC)	
NEC - Bell's stage 2 or greater	◯ Yes ◯ No
If yes:	☐ Medically Treated<br>☐ Surgically Treated
Sepsis	
Early Onset Sepsis (< 72 hrs of age) -requiring treatment:	◯ Yes ◯ No
Body fluid:	☐ Blood ☐ CSF ☐ Urine<br>(check all that apply)
Late Onset Sepsis (>/= 72 hrs of age) - requiring treatment:	◯ Yes ◯ No
Body fluid:	☐ Blood ☐ CSF ☐ Urine<br>(check all that apply)
Respiratory	
Did infant require mechanical ventilation?	◯ Yes ◯ No
Duration of conventional Mechanical Ventilation (CMV)	__________________________<br>(days)
Duration of High Frequency Ventilation (HFV)	__________________________<br>(days)

Did infant require inhaled nitric oxide (iNO)?	◯ Yes ◯ No
Duration iNO	____________ (hours)
Did infant require non-invasive ventilation/pressure support or oxygen therapy?	◯ Yes ◯ No
Duration of CPAP (including nasal IPPV or biphasic)	____________ (days)
Duration of High Flow Nasal Cannula:	____________ (days)
Duration of Low Flow Nasal Cannula:	____________ (days)
Respiratory Complications	☐ Pulmonary hemorrhage<br>☐ Pulmonary hypertension<br>☐ Pulmonary interstitial emphysema<br>☐ Other<br>☐ None<br>(check all that apply)
If other:	____________
Was there a radiologic diagnosis of pulmonary air leak including pneumothorax, pneumomediastinum or pulmonary interstitial emphysema (PIE) during the NICU stay?	◯ Yes ◯ No<br>((minimal dataset))
If yes, was it diagnosed within 48 hours of delivery?	◯ Yes ◯ No<br>((minimal dataset))
If yes complete REDCap Adverse Event form	
Postnatal steroids for treatment of BPD (Bronchopulmonary Dysplasia)	◯ Yes ◯ No
Number of courses	◯ 1 ◯ 2 ◯ 3
1st Course	
1st course start date:	____________ (dd-mm-yyyy)
1st course end date:	____________ (dd-mm-yyyy)
2nd Course	

2nd course start date: ____________________ (dd-mm-yyyy)

2nd course end date: ____________________ (dd-mm-yyyy)

3rd Course

3rd course start date: ____________________ (dd-mm-yyyy)

3rd course end date: ____________________ (dd-mm-yyyy)

Day 28

Infant status at 28 days
- ◯ Died
- ◯ Remains in NICU/Special Care/Level II Nursery
- ◯ Yes - transferred
- ◯ Unknown - Transfer, further data unavailable

Please fill out REDCap Adverse Event [AE] form AND Complete an SAE report form and send to LCC within 48 hrs of investigator becoming aware of death

Respiratory support at 28 days
- ◯ None
- ◯ Mechanical ventilation
- ◯ NIPPV
- ◯ CPAP
- ◯ HFNC >2 L/min
- ◯ NC ≤2L/min
- ◯ Missing/unknown

Oxygen therapy at 28 days ◯ Yes ◯ No ◯ Missing/unknown

FiO2 route ◯ FiO2 ◯ Flow rate if LFNC

FiO2 ____________________

Flow rate if LNFC ____________________ (L/min)

36 Weeks / Discharge

Infant status at 36 weeks	◯ Died<br>◯ Remains in NICU/Special Care/Level II Nursery<br>◯ Yes - discharged/transferred<br>◯ Discharged home<br>◯ Unknown - Transfer, further data unavailable
Please fill out REDCap Adverse Event [AE] form AND Complete an SAE report form and send to LCC within 48 hrs of investigator becoming aware of death	
Head Circumference at 36 weeks or last recorded value prior to discharge home (cm)	__________________<br>(cm)
	☐ Head circumference missing/unknown
Weight at 36 weeks or last recorded weight prior to discharge home (g)	__________________<br>(grams)
	☐ Weight missing / unknown
Respiratory support at 36 weeks / at discharge home	◯ None<br>◯ Mechanical ventilation<br>◯ NIPPV<br>◯ CPAP<br>◯ HFNC >2 L/min<br>◯ NC ≤2L/min<br>◯ Other<br>◯ Missing/unknown
If other:	__________________
Oxygen therapy at 36 weeks / at discharge home	◯ Yes ◯ No ◯ Missing/unknown
FiO2 route	◯ FiO2 ◯ Flow rate if LFNC
FiO2	__________________
Range is outside of (range 0.22-1.0) Please check data entry.	
Flow rate if LFNC	__________________<br>(L/min)
Range is outside of (range 0.01-2.0) Please check data entry.	

40 Weeks or / Discharge

Infant status at 40 weeks	◯ Died<br>◯ Remains in NICU/Special Care/Level II Nursery,<br>◯ Yes - discharged/transferred<br>◯ Discharged home<br>◯ Unknown - Transfer, further data unavailable

Please fill out REDCap Adverse Event [AE] form AND Complete an SAE report form and send to LCC within 48 hrs of investigator becoming aware of death

Respiratory support at 40 weeks / at discharge home	◯ None<br>◯ Mechanical ventilation<br>◯ NIPPV or biphasic<br>◯ CPAP<br>◯ HFNC >2 L/min<br>◯ NC ≤2L/min<br>◯ Other<br>◯ Missing/unknown
If other:	______________________
Oxygen therapy at 40 weeks / at discharge home	◯ Yes ◯ No ◯ Missing/unknown
FiO2 route	◯ FiO2 ◯ Flow
FiO2	______________________

Range is outside of (range 0.22-1.0) Please check data entry.

Flow	______________________<br>(L/min)

Range is outside of (range 0.01-2.0) Please check data entry.

End Of NICU Stay

Consent not obtained - collect minimal dataset only

Infant not eligible or parents declined consent

Section G: End of NICU Stay

Disposition at end of NICU/Special Care/Level II Nursery stay:

- ◯ Discharged Home
- ◯ Transferred to pediatric ward
- ◯ Remains in NICU at 48 weeks PMA
- ◯ Transfer out of study center, unable to obtain further data
- ◯ Died prior to NICU discharge

((minimal dataset))

[disposition]: Date

____
(dd-mm-yyyy(minimal dataset))

Did the infant die during NICU admission:

◯ Yes ◯ No
((minimal dataset))

If yes complete an Adverse Event form in REDCap AND complete an SAE report form and send to LCC within 48 hrs of investigator becoming aware of death)

Date of Death

____
(dd-mm-yyyy(minimal dataset))

Primary cause of death

- ◯ Pulmonary hypoplasia
- ◯ Severe RDS
- ◯ CLD
- ◯ Pneumonia
- ◯ Gr 3 or 4 IVH
- ◯ Meningitis
- ◯ Septicemia
- ◯ NEC
- ◯ Sudden unexplained death
- ◯ Other

((minimal dataset)choose one)

Primary cause of death - Other (describe)

____
((minimal dataset))

Secondary cause of death	☐ Pulmonary hypoplasia<br>☐ Extreme Prematurity<br>☐ Severe RDS<br>☐ CLD<br>☐ Pneumonia<br>☐ Gr 3 or 4 IVH<br>☐ Meningitis<br>☐ Septicemia<br>☐ NEC<br>☐ Sudden unexplained death<br>☐ Other<br>((minimal dataset)choose all that apply)
Secondary cause of death - Other (describe)	______________________<br>((minimal dataset))
Last weight obtained:	______________________<br>(g(minimal dataset))
	☐ Weight Missing / unknown
On oxygen therapy at disposition?	◯ Yes ◯ No<br>((minimal dataset))

Congenital Abnormalities

Consent not obtained - collect minimal dataset only (no fields on this form)

Infant not eligible or parents declined consent

Congenital Abnormalities

Were there any congenital or chromosomal abnormalities identified during the NICU stay	◯ Yes ◯ No
If yes, describe	__________________________

Co-enrolment

Consent not obtained - collect minimal dataset only (no fields on this form)

Infant not eligible or parents declined consent

Section I: Co-enrolment

Was infant co-enrolled in other research trials? ◯ Yes ◯ No

List co-enrolled trials: ______________________________

Long-term neurodevelopmental outcomes (18-24months)

Consent not obtained - collect minimal dataset only (no fields on this form)

Infant not eligible or parents declined consent

Infant died in NICU prior to discharge.

Long-term neurodevelopmental outcomes (18-24months)

Did the infant have 2 year follow up assessment:	◯ Yes ◯ No
If no, state reason:	◯ Died after NICU stay<br>◯ Lost to follow-up<br>◯ Parents declined followup
If died after NICU stay, last known date alive:	______________________<br>(dd-mm-yyyy)
If Yes, Type of contact:	◯ Clinic Visit ◯ Phone Call only
If assessment completed, Date of assessment:	______________________<br>(dd-mm-yyyy)
Does child have vision impairment:	◯ Yes ◯ No
Type of vision impairment:	◯ Unilateral ◯ Bilateral
Outcome of vision impairment:	◯ Functional with corrective lenses<br>◯ Blind, some functional vision<br>◯ Blind, no useful vision
Does child have hearing impairment:	◯ Yes ◯ No
type of hearing impairment:	◯ Unilateral ◯ Bilateral
Outcome of hearing impairment:	◯ Has some hearing problems but does NOT need a hearing aid<br>◯ Hears well or with only a little difficulty WITH a hearing aid<br>◯ Has severe hearing difficulty with a hearing aid or hearing is not helped with an aid
Has this child been diagnosed with cerebral palsy:	◯ Yes ◯ No
If Yes, is the CP	◯ Mild (unsteady walk)<br>◯ Moderate (unable to walk without help)<br>◯ Severe (cannot walk)<br>◯ Missing / unknown

Gross Motor Function Classification System (GMFCS) scale:

◯ 1 ◯ 2 ◯ 3 ◯ 4 ◯ 5 ◯ Unknown

Does this child have problems with language or speech: ◯ Yes ◯ No

Does this child use more than 10 recognizable words: ◯ Yes ◯ No

(includes signed words)

Was the infant admitted to the Hospital since discharge? ◯ Yes ◯ No ◯ Missing

Number of hospital admissions

◯ 1
◯ 2
◯ 3
◯ 4
◯ 5

Hospital admission 1
Reason for admission ______________________

Hospital admission 2
Reason for admission ______________________

Hospital admission 3
Reason for admission ______________________

Hospital admission 4
Reason for admission ______________________

Hospital admission 5
Reason for admission ______________________

Respiratory Medications since discharge: ◯ Yes ◯ No

If Yes, list what medications: ____________________________

Does this child have any major medical problems or diagnosis: ◯ Yes ◯ No

If Yes, please describe: ____________________________

ASQ 3 (Ages and Stages Questionnaires)

Assessment complete	◯ Yes ◯ No
If No, reason not complete:	______________________
Date ASQ-3 completed:	______________________ (dd-mm-yyyy)
Communication total score:	______________________
Gross motor total score:	______________________
Fine motor total score:	______________________
Problem solving total score:	______________________
Personal-social total score:	______________________

BSID IV (Bayley Scales of Infant and Toddler Development Edition IV)

Assessment complete	◯ Yes ◯ No
If No, reason not complete:	______________________
Date BSID IV completed	______________________ (dd-mm-yyyy)
Cognitive	
Cognitive raw score	______________________
Cognitive scaled score	______________________
Cognitive composite score	______________________
Language	
Language Raw score	
Receptive communication	______________________

Expressive communication

____

Language Scaled score

Receptive communication

____

Expressive communication

____

Language Sum

____

Language Composite Score

____

Motor

Motor raw score

Fine motor

____

Gross motor

____

Motor scaled score

Fine motor

____

Gross motor

____

Motor Sum

____

Motor composite score

____

Social-emotional

Social-emotional raw score

____

Social-emotional scaled score

____

Social emotional composite score

____

End Of Study

Infant not eligible or parents declined consent

Complete this form once all data for this patient has been collected and entered into REDCap, including Adverse Events and Protocol Deviations (if applicable) to allow for data locking.

End of Study Status

◯ Minimal data set only (unable to obtain parental consent)
◯ Enrolled (consented) & follow-up completed
◯ Enrolled (consented) and lost to follow-up
◯ Enrolled & parents withdrew consent
◯ Enrolled and died before follow-up

Study End Date

____

Please enter date of NICU discharge or death as the End of Study date.

Please enter date of follow-up visit as the End of Study date.

Please enter date lost to follow-up known as the End of Study date.

Please enter date of consent withdrawal as the End of Study date.

Please enter last known date alive as the End of Study date.

Comments

____

Adverse Events

Infant not eligible or parents declined consent

Use this form to record details of any adverse event that occurs during the course of the study.

Note that a worsening of a pre-existing condition is also an adverse event.

Add additional instances for each Adverse Event.

Did an Adverse Event occur?	◯ Yes ◯ No<br>((minimal dataset))
IF yes, which adverse event occurred?	◯ a. Pulmonary air leak in 1st 48 hours<br>◯ b. Cardiac compressions in the delivery room<br>◯ c. Grade III or IV intraventricular hemorrhage<br>◯ d. Any condition leading to death in the delivery room<br>◯ e. Any condition leading to death in the NICU<br>◯ f. Any other event deemed to be an AE by Site Investigator<br>((minimal dataset))

For events c, d, & e (Protocol defined SAE's):
Complete a REDCap Adverse Event [AE] form
AND
Complete a Serious Adverse Event report form and send to LCC within 48 hours of investigator becoming aware of event)

Description of AE:	____________________________<br>((minimal dataset))
Reported Term for Adverse Event	________________________<br>(please use diagnosis when possible(minimal dataset))
Severity of AE?	◯ Mild<br>◯ Moderate<br>◯ Severe<br>◯ Life-threatening<br>◯ Death<br>((minimal dataset))
Attribution to HiLo intervention:	◯ Unrelated<br>◯ Unlikely<br>◯ Possibly<br>◯ Probably<br>◯ Definitely<br>((minimal dataset))
Serious (SAE)?	◯ Yes ◯ No

Start Date	______________________<br>((minimal dataset))
Start Time	______________________<br>((minimal dataset))
Outcome	◯ Recovered / Resolved With No Sequelae<br>◯ Recovering / Resolving<br>◯ Not Recovered / Unresolved<br>◯ Recovered / Resolved With Sequelae<br>◯ Fatal<br>((minimal dataset))
Stop Date	______________________<br>((minimal dataset))
Stop Time	______________________<br>((minimal dataset))

Adverse Event Coding

Use this form to code data that has been entered on the Adverse events form. Use one instance of this form for each reported event.

A verbatim adverse event (AE) description may contain multiple medical concepts (for example diarrhea and vomiting) rather than a simple diagnosis. A single MedDRA term therefore may not adequately reflect the AE description. This form includes multiple code fields for each AE so that more than one MedDRA term can be associated with each AE description.

Start typing text in the box to search for an appropriate term. Use an asterisk '*' to represent wild cards where necessary.

To view the MedDRA terms within the context of the MedDRA hierarchy use the MedDRA web browser.

Note: Codes entered on this form may be invalidated if the adverse event record is changed. Coding on this form should only be performed on locked AE forms immediately prior to analysis. A full quality review of exported adverse event data should be performed immediately prior to database lock.

MedDRA is licensed for not-for-profit use by the University of Alberta Libraries.

There is no adverse event instance [current-instance] to code.

[ae_description][current-instance]
[ae_term][current-instance]

Start Date: [ae_startd][current-instance]
Serious: [ae_serious][current-instance]

MedDRA term	______________________
Add another term?	☐ Yes
MedDRA term	______________________
Add another term?	☐ Yes
MedDRA term	______________________

Protocol Deviations

Infant not eligible or parents declined consent

Did a protocol deviation occur?	◯ Yes ◯ No<br>((minimal dataset))
If Yes:	◯ Consent procedures (wrong consent version used; consent form missing; date/signatures missing)<br>◯ AE reporting (Not reporting within 48 hrs; not reporting to IRB)<br>◯ Wrong oxygen concentration used at start of resuscitation<br>◯ HiLo delivery algorithm not followed during resuscitation<br>◯ Surfactant given in the first 10 minutes of life<br>◯ Other<br>((minimal dataset))
Category of "Other"	__________________________________
Description of protocol deviation:	____<br>((minimal dataset))
Date Deviation Occurred	__________________________________<br>((minimal dataset) dd-mm-yyyy)
Date IRB Notified (if applicable):	__________________________________<br>((minimal dataset) dd-mm-yyyy)

Unanticipated Problems

Infant not eligible or parents declined consent

Did an Unanticipated Problem occur? ◯ Yes ◯ No
((minimal dataset))

1. Date UP Identified: ______________________
((minimal dataset))

2. Identify UP: ______________________
((minimal dataset) Give the UP a brief title.)

3. The Unanticipated Problem was unexpected in terms of nature, severity or frequency: ◯ Yes ◯ No
((minimal dataset))

4. The Unanticipated Problem is possibly related to participation in the research: ◯ Yes ◯ No
((minimal dataset))

5. The Unanticipated Problem suggests that the research places subjects or others at a greater risk of harm than was previously known or recognized: ◯ Yes ◯ No
((minimal dataset))

The answers to questions 3-5 are ALL "YES", report event as an Unanticipated Problem as applicable.

6. Briefly Describe the UP (Include additional or supplementary information as necessary. Include date of incident, date of discovery, describe harm or potential harm that occurred to subject(s), whether the incident is resolved, whether the subject(s) remains on study):
((minimal dataset))

7. What action was taken with the study as a result of the Unanticipated Problem? (Check all that apply.)
((minimal dataset))

☐ No action
☐ Revise protocol to eliminate apparent immediate hazards to subjects
☐ Modification of inclusion or exclusion criteria to mitigate newly identified risks
☐ Implementation of additional procedures for monitoring subjects
☐ Suspension of enrollment of new subjects
☐ Notify currently enrolled subjects
☐ Modification of consent documents to include a description of newly recognized risks (site and/or study wide)
☐ Provision of additional information about newly recognized risks to previously enrolled subjects
☐ Other:

Describe other action ______________________
((minimal dataset))

8. Is the Unanticipated Problem a serious adverse event? ◯ Yes ◯ No
((minimal dataset))

*If the Unanticipated Problem is a serious adverse event, submit this form and make sure that the adverse event form and Serious Adverse Event report have been completed and submitted as per local site policy.

Data Management Tracking

For use by the Data Coordinating Centre	
Data management complete?	◯ Yes ◯ No
Date DM completed	__________________________________<br>(Optional, enter once complete.)
Participant forms locked?	◯ Yes ◯ No
Date participant locked.	__________________________________<br>(Optional, enter once locked.)
Flagged for Monitoring	☐ SDV
Notes	
Files	

Mark this form as complete only once all data management work has been completed AND the participant locked.

Investigator Signoff

By selecting this box and applying my REDCap electronic signature to this form, I confirm that I am the Qualified Investigator (QI) for this study site. I have reviewed all electronic data for this participant and find it to be a true and accurate representation of the participant's records as required by the study protocol.	☐ Reviewed and Approved
