# Supplementary material for: Does the use of higher versus lower oxygen concentration improve neurodevelopmental outcomes at 18–24 months in very low birthweight infants?
Source: Trials. 2024 Apr 4;25:237. doi: 10.1186/s13063-024-08080-2 (PMC10996184; doi:10.1186/s13063-024-08080-2)
Supplement: Supplementary file 1 — Supplementary Material 1. [file 13063_2024_8080_MOESM1_ESM.zip › View Protocol Section NCT03825835R2.pdf]

View Protocol Section

Study Identification

Unique Protocol ID: Pro00083931

Brief Title: 30% or 60% Oxygen at Birth to Improve Neurodevelopmental Outcomes in Very Low Birthweight Infants (HiLo)

Official Title: Does the Use of Higher Versus Lower Oxygen Concentration Improve Neurodevelopmental Outcomes at 18-24 Months in Very Low Birthweight Infants - The HiLo-Trial

Secondary IDs:

Study Status

Record Verification: March 2024

Overall Status: Recruiting

Study Start: June 27, 2022 [Actual]

Primary Completion: September 30, 2027 [Anticipated]

Study Completion: December 30, 2027 [Anticipated]

Sponsor/Collaborators

Sponsor: University of Alberta

Responsible Party: Sponsor

Collaborators: University of Toronto
University of Sydney
University of Valencia
Dalhousie University
McMaster University
University of Manitoba
McGill University
University of Calgary
Memorial University of Newfoundland
University College Cork
University of British Columbia
Laval University

Université de Montréal
University of Ottawa

Oversight

U.S. FDA-regulated Drug: No

U.S. FDA-regulated Device: No

U.S. FDA IND/IDE: No

Human Subjects Review: Board Status: Approved Approval Number: Pro00083931
Board Name: Health Ethics Reserach Board
Board Affiliation: University of Alberta
Phone: 780-492-0459 Email: reoffice@ualberta.ca
Address:
Research Ethics Office
116 St & 85 Ave, Edmonton, AB T6G 2R3
Canada

Data Monitoring: Yes

FDA Regulated Intervention: No

Study Description

Brief Summary:

Preterm birth, or birth before 37 weeks' gestation, is increasingly common, occurring in 8 percent of pregnancies in Canada. Preterm birth is associated with many health complications, particularly when the birth happens before 29 weeks' gestation. At this gestational age, the lungs are not fully developed and it is not uncommon for infants to have problems breathing at the time of birth. One complication that can arise is when an infant stops breathing and needs to be resuscitated. When preterm babies need to be resuscitated doctors must take special care because of the small infant size and the immaturity of the brain and lungs. Oxygen is used to resuscitate babies who need it, but unfortunately there is disagreement about the best oxygen concentration to use. Oxygen concentration is important because both too much and too little oxygen can cause brain injury. This research aims to fill this knowledge gap by participating in an international clinical trial to compare the effects of resuscitating babies less than 29 weeks' gestational age with either a low oxygen concentration or a high oxygen concentration. The oxygen concentrations have been selected using the best available knowledge.

This will be a cluster randomized trial where each participating hospital will be randomized to either 30 or 60 percent oxygen for the recruitment of 30 infants, and afterwards randomized to the

other group for the recruitment of another 30 infants. After the trial, the investigator will determine whether the babies resuscitated with low oxygen or those resuscitated with high oxygen have better survival and long-term health outcomes. This research fills a critical knowledge gap in the care of extremely preterm babies and will impact their survival both here in Canada and internationally.

Detailed Description:

Purpose: Over the last 10 years, recommendations regarding the ideal level of oxygen for resuscitation in preterm infants have changed from 100 percent, down to low levels of oxygen (<30 percent), up to moderate concentration (30-65 percent). In addition, in 2010, oxygen saturation targeting was recommended as standard of care and this contributed to a change in clinical practice as clinicians were more likely and comfortable to start resuscitation at either 21percent (room air) or titrated levels of oxygen such as 30-40 percent. When the guidelines were again revised in 2015, the International Liaison Committee on Resuscitation (ILCOR) acknowledged that a critical knowledge gap continued to exist for the resuscitation of the preterm infants <37 weeks, highlighting the need to provide more concrete guidelines. This leaves clinicians in a challenging position. Despite the advances that have been achieved in perinatal and neonatal care, neonates are still vulnerable to the consequences of the oxidative effects from hyperoxia as well as the deleterious effects from hypoxia. A large, multi-centre international trial of sufficient sample size that is powered to look at safety outcomes such as mortality and adverse neurodevelopmental outcomes is required to provide the necessary evidenced to guide clinical practice with confidence.

Hypothesis: the null hypothesis for this study is that the incidence of mortality or abnormal neurodevelopmental outcomes at 18-24 months corrected age will be no different by using either higher initial oxygen concentration of 60 percent compared to using lower initial oxygen concentration of 30 percent for resuscitation of preterm infants of 23 0/7- 28 6/7 weeks gestation.

Justification:

The use of supplementary oxygen may be crucial, but also potentially detrimental to premature infants at birth. High oxygen levels may lead to organ damage through oxidative stress, while low oxygen levels may lead to increased mortality. Excess oxygen exposure during the early post-birth period is associated with many complications and morbidities of preterm birth. Preterm infants have lower levels of anti-oxidant pathways consistent with their

expected fetal environment of low oxygen exposure. Excess of oxygen free-radicals in infants intrinsically deficient in enzymatic antioxidants and non-enzymatic antioxidants may contribute to these morbidities. Pulmonary oxygen toxicity, through the generation of reactive oxygen and nitrogen species in excess of antioxidant defenses, is believed to be a major contributor to the development of bronchopulmonary dysplasia (BPD). Using lower oxygen concentrations at birth results in decreased oxidative stress markers and a decrease risk of developing BPD compared to higher oxygen concentrations. Other organs that may be damaged by such oxidative stress include kidneys, myocardium and the retina.

There is equally growing evidence that using lower oxygen concentrations will lead to lower oxygen saturation levels and bradycardia, which may lead to increased rates of mortality in this vulnerable group of infants. An individual patient analysis of clinical trials reported that 46% of preterm infants resuscitated with initial low oxygen concentration did not reach SpO2 of 80% at 5 min. This was associated with increased risk of major intraventricular hemorrhage (IVH), and an almost five times higher risk of death in this vulnerable group of infants. These data provide a warning note for the use of higher vs. lower initial oxygen concentration during delivery room resuscitation. As the investigator proceed in determining a safe range for resuscitation of ELBW/ELGA infants, it is highly likely that the optimum level of oxygen concentration is between the two extremes of 21 percent and 100 percent.

Objectives: To determine whether initial resuscitation of preterm neonates with 60 percent versus 30 percent oxygen results in better neurodevelopmental outcomes at 18-24 months.

Research Method/Procedures: This will be a cluster crossover design, unmasked randomized controlled trial (RCT) comparing two oxygen concentrations at initiation of resuscitation. Infants will be placed on the resuscitation table with the initial steps of resuscitation carried out as per standard of care at each centre which usually follows current resuscitation guidelines. All centres will make every effort to establish adequate lung expansion using CPAP or positive pressure ventilation as needed. Enrolled infants will have a pulse oximeter sensor placed on the right arm in the first minute of life. Their resuscitation will be initiated with an oxygen concentration of 30 or 60 percent depending on the randomization sequence at the centre at the given time. Infants in the 30 percent group will remain in 30 percent oxygen until 5 min of age unless the infant's heart rate (HR) remains 100/min or less and does not show a tendency towards progressive increase before

reaching 5 min of age or infant needs chest compression and/or epinephrine. No alteration in oxygen concentration will be made for an infant who is responding to resuscitation efforts with HR progressively increasing as minutes go by. At 5 min of age, the clinical team will assess oxygen saturation. If the saturation is less than 85 percent, oxygen should be increased by 10-20 percent every 60 sec to achieve saturations of 85 percent or greater or a saturation of 90-95 percent at 10 min of age. If saturations are greater than 95 percent at or before 5 min of age, oxygen should be decreased stepwise (every 60 sec) with an aim to maintain saturations of 85 percent or greater during 5-10 min of age or 90-95 percent at and beyond 10 min of age. The procedure for infants in the 60 percent group will be identical. The intervention duration for the trial will be the first 5 min after birth followed by initial monitoring/action for the next 5 min where titration in oxygen concentration will be made to achieve stability making a total of 10 min for study intervention. Titration of oxygen before 5 min after birth will only be made if the infant remains bradycardic (HR less than 100) and does not show a tendency towards a sustained increase in HR or if the oxygen saturation exceeds 95 percent. If the infant does not respond to ventilation with increasing HR in the first 5 min after birth, steps to ensure effective ventilation should be done before oxygen is titrated.

Plan for Data Analysis: Generalized linear mixed model with binary outcome and maximum likelihood estimate will be used to evaluate the effect of an oxygen concentration on the primary outcome (as a composite between 18 and 24 months corrected age of all-cause mortality or the presence of a major neurodevelopmental outcome). To account for cluster crossover design of the study, effects of centers (clusters) and a period (oxygen concentration) within center will be considered random, and effects of a period (oxygen concentration) will be entered as a fixed effect. This hierarchical model allows for the correlation of patients within periods and within clusters. The model will be adjusted for gestational age and whether or not infant required mask ventilation as potential confounding variables. Similar generalized linear mixed models will be performed to evaluate the effect of group on secondary outcomes. In addition, three subgroup analysis will be performed: i) Gestational age will be categorized into 2 categories: 23+0- 25+6 vs. 26+0-28+6 weeks; ii) Breathing support will be categorized by infants supported only with CPAP vs. received mask ventilation; iii) Sex/Gender will be categorized into 2 categories: female vs. male. For subgroup analysis baseline characteristics will be compared using linear and generalized linear mixed models. Sensitivity analysis will be performed to analyze the missing data; however, a very low number of missing values are

expected due to the design of the study.

Conditions

Conditions: Premature Infant
Respiratory Distress Syndrome in Premature Infant
Neurodevelopmental Outcome

Keywords: Delivery Room
Resuscitation
Premature Infant
Oxygen

Study Design

Study Type: Interventional
Primary Purpose: Prevention
Study Phase: N/A
Interventional Study Model: Parallel Assignment
Number of Arms: 2
Masking: Single (Outcomes Assessor)

Outcome assessed will be unaware of group allocation

Arms and Interventions

Arms	Assigned Interventions
Active Comparator: 30% group<br><br>Infants in the 30% oxygen group will remain in 30% oxygen (O2) until 5 min of age. At 5 min of age, the clinical team will assess oxygen saturation (SpO2). If SpO2 is <85%, O2 should be increased by 10-20% every 60 sec to achieve SpO2 of 85% or greater or a SpO2 of 90-95% at 10 min of age. If SpO2 are greater than 95% at or before 5 min of age, O2 should be decreased stepwise (every 60 sec) with an aim to maintain SpO2 of 85% or greater	Drug: 30% oxygen group<br><br>Infants in the 30% oxygen group will remain in 30% oxygen (O2) until 5 min of age. At 5 min of age, the clinical team will assess oxygen saturation (SpO2). If SpO2 is <85%, O2 should be increased by 10-20% every 60 sec to achieve SpO2 of 85% or greater or a SpO2 of 90-95% at 10 min of age. If SpO2 are greater than 95% at or before 5 min of age, O2 should be decreased stepwise (every 60 sec) with an aim to maintain SpO2 of 85% or greater

during 5-10 min of age or 90-95% at and beyond 10 min of age.<br><br>Intervention: Infants randomized to the 30% oxygen group will receive 30% oxygen at birth for the first 5 minutes. At 5 minutes oxygen can be adjusted as needed.	during 5-10 min of age or 90-95% at and beyond 10 min of age.<br><br>Intervention: Infants randomized to the 30% oxygen group will receive 30% oxygen at birth for the first 5 minutes. At 5 minutes oxygen can be adjusted as needed.
Experimental: 60% group<br><br>Infants in the 60% oxygen group will remain in 60% oxygen (O2) until 5 min of age. At 5 min of age, the clinical team will assess oxygen saturation (SpO2). If SpO2 is <85%, O2 should be increased by 10-20% every 60 sec to achieve SpO2 of 85% or greater or a SpO2 of 90-95% at 10 min of age. If SpO2 are greater than 95% at or before 5 min of age, O2 should be decreased stepwise (every 60 sec) with an aim to maintain SpO2 of 85% or greater during 5-10 min of age or 90-95% at and beyond 10 min of age.<br><br>Intervention: Infants randomized to the 60% oxygen group will receive 60% oxygen at birth for the first 5 minutes. At 5 minutes oxygen can be adjusted as needed.	Drug: 60% oxygen group<br><br>Infants in the 60% oxygen group will remain in 60% oxygen (O2) until 5 min of age. At 5 min of age, the clinical team will assess oxygen saturation (SpO2). If SpO2 is <85%, O2 should be increased by 10-20% every 60 sec to achieve SpO2 of 85% or greater or a SpO2 of 90-95% at 10 min of age. If SpO2 are greater than 95% at or before 5 min of age, O2 should be decreased stepwise (every 60 sec) with an aim to maintain SpO2 of 85% or greater during 5-10 min of age or 90-95% at and beyond 10 min of age.<br><br>Intervention: Infants randomized to the 60% oxygen group will receive 60% oxygen at birth for the first 5 minutes. At 5 minutes oxygen can be adjusted as needed.

NOTE: Intervention Other Names have not been specified
NOTE: Intervention Other Names have not been specified

Outcome Measures

Primary Outcome Measure:

1. Neurodevelopmental outcome at 18-24 months

   At 18-24 months corrected age, the infants will be assessed using the Bayley Scales of Infant Development-3rd edition (Bayley-III). A score 85-114 will be classified as normal, and scores <70 (2 SD below the mean of 100) will define severe cognitive delay.

   [Time Frame: 18-24 months of age]

2. Healing loss

   Audiometry will be performed to assess the presence or absence of severe hearing loss.

   [Time Frame: 18-24 months of age]
3. Blindness

   Blindness will be defined as a corrected visual acuity of <20/200.

   [Time Frame: 18-24 months of age]

Secondary Outcome Measures:

4. Number of intubation in the delivery room

   Number of infants intubated in the delivery room

   [Time Frame: first 15 minutes after birth]
5. Death in the Neonatal Intensive Care Unit

   Number of infants died after admission in the Neonatal Intensive Care Unit

   [Time Frame: During admission in the Neonatal Intensive Care Unit; no exact time frame can be given as this can happen after 1 day, 1 week, 1 months or up to 2-3 month. again deepening on gestational age and policy for transferring infants at participating centres.]
6. Death in the delivery room

   Number of infants died in the delivery room during resuscitation

   [Time Frame: During resuscitation in the delivery room; for lay people: this time frame might be 10min or 1-3 hours, depending on the approaching each participating hospital]

Eligibility

Minimum Age: 0 Minutes

NOTE: Minimum Age '0 Minutes' is treated as no limit.

Maximum Age: 10 Minutes

Sex: All

Gender Based: No

Accepts Healthy Volunteers: No

Criteria:

Inclusion Criteria:

- Infants born at 23 0/7 weeks to 28 6/7 weeks' gestational age who will receive full resuscitation and are without major congenital abnormalities

Exclusion Criteria:

- Infants who are outborn - initial resuscitation not performed at the study centre
- Infants who are not born within the eligible gestational age range- this trial is specific to preterm infants
- Infants who are born with a major congenital abnormality- congenital abnormalities may affect oxygenation or neurodevelopmental outcomes
- Infants who will not receive full resuscitation at birth- these infants will not receive resuscitation

Contacts/Locations

Central Contact Person: Georg Schmolzer, MD, PhD
Telephone: 7807354647
Email: schmolze@ualberta.ca

Central Contact Backup: Barb Kamstra, RN
Telephone: 7807354647
Email: Barb Kamstra <Barbara.Kamstra@albertahealthservices.ca>

Study Officials: Georg Schmolzer, MD, PhD
Study Principal Investigator
University of Alberta

Locations:

Canada, Alberta

Royal Alexandra Hospital — Active, not recruiting

Edmonton, Alberta, Canada, T5H 3V9

Contact: Georg SCHMOLZER 7807354660 georg.schmoelzer@me.com

Principal Investigator: Georg Schmolzer

Sub-Investigator: Brenda Law

Canada, Ontario

Sunnybrook Health Sciences Centre — Active, not recruiting

Toronto, Ontario, Canada
Contact: Elizabeth Asztalos elizabeth.asztalos@sunnybrook.ca
Contact: Afsheen Ayaz afsheen.ayaz@sunnybrook.ca
Principal Investigator: Elizabeth Asztalos

Canada, Alberta

Foothills Hospital — Active, not recruiting
Calgary, Alberta, Canada
Contact: Amuchou Soraisham Amuchou.Soraisham@albertahealthservices.ca
Principal Investigator: Amuchou Soraisham

Canada, Ontario

Neonatal Intensive Care Unit - Mount Sinai Hospital — Active, not recruiting
Toronto, Ontario, Canada
Contact: Prakesh SHAH Prakeshkumar.Shah@sinaihealth.ca
Principal Investigator: Prakesh SHAH

Neonatal Intensive Care Unit - Hamilton Health Sciences — Recruiting
Hamilton, Ontario, Canada, L8S 4K1
Contact: Amit MUKERJI mukerji@mcmaster.ca
Principal Investigator: Amit MUKERJI

Canada, Nova Scotia

Newborn Health - IWK Health Centre — Recruiting
Halifax, Nova Scotia, Canada
Contact: Walid EL-NAGGAR Walid.El-Naggar@iwk.nshealth.ca
Principal Investigator: Walid EL-NAGGAR

Canada, Quebec

McGill Univeristy — Recruiting
Montréal, Quebec, Canada
Contact: Marc Beltempo marc.beltempo@mcgill.ca
Contact: Francois Olivier francois.olivier@mcgill.ca

Principal Investigator: Marc Beltempo
Principal Investigator: Francois Olivier

Canada, Manitoba

Health Sciences Not yet recruiting
Winnipeg, Manitoba, Canada
Contact: Ayman Sheta asheta@hsc.mb.ca
Principal Investigator: Ayman Sheta

Canada, NL

Janeway Children's Health and Rehabilitation Centre Recruiting
Saint John's, NL, Canada, A1B 3V6
Contact: Jo-Anna Hudson, MD

Canada, British Colubia

BC Children Not yet recruiting
Vancouver, British Colubia, Canada
Contact: Sandesh Shivananda sandesh.shivananda@cw.bc.ca

Canada, Ontario

CHEO Not yet recruiting
Ottawa, Ontario, Canada
Contact: Laurent Renesme LRenesme@cheo.on.ca

Canada, Quebec

Chu University Laval Recruiting
Québec, Quebec, Canada
Contact: Christine Drolet christine.drolet.med@ssss.gouv.qc.ca

Ireland

University College Cork Not yet recruiting
Cork, Ireland
Contact: Eugene DEMPSEY G.Dempsey@ucc.ie

Spain

Hospital Universitario Dexeus — Not yet recruiting
Barcelona, Spain
Contact: Hector Boix Alonso hector.boix@quironsalud.es

Hospital de Asturias — Not yet recruiting
Oviedo, Spain
Contact: Hector Boix Alonso hector.boix@quironsalud.es

Hospital Germans Tries i Pujol — Not yet recruiting
Barcelona, Spain
Contact: Hector Boix Alonso hector.boix@quironsalud.es

Hospital Universitario Materno Infantil Miguel Servet — Not yet recruiting
Zaragoza, Spain
Contact: Hector Boix Alonso hector.boix@quironsalud.es

Hospital de la Arrixaca — Not yet recruiting
El Palmar, Spain
Contact: Hector Boix Alonso hector.boix@quironsalud.es

Hospital Las Palmas — Not yet recruiting
Las Palmas De Gran Canaria, Spain
Contact: Hector Boix Alonso hector.boix@quironsalud.es

NOTE: Locations have 6 notes.

IPD Sharing Statement

Plan to Share IPD: Yes

The data will be included in a prospectively planned IPD, which is called PROMOTION Other researchers can email the PI for more information at georg.schmoelzer@me.com

Supporting Information: Study Protocol
Statistical Analysis Plan (SAP)
Informed Consent Form (ICF)
Clinical Study Report (CSR)
Analytic Code

Time Frame:
after the publication of the primary results indefinitely

Access Criteria:
Other researchers can email the PI for more information at georg.schmoelzer@me.com

URL:

References

Citations:

Links:

Available IPD/Information:

Record Summary

U.S. National Library of Medicine | U.S. National Institutes of Health | U.S. Department of Health & Human Services | HHS Vulnerability Disclosure
